# Deconstructed Plastic Substrate Preferences of Microbial Populations from the Natural Environment

**DOI:** 10.1128/spectrum.00362-23

**Published:** 2023-06-01

**Authors:** Lindsay I. Putman, Laura G. Schaerer, Ruochen Wu, Daniel G. Kulas, Ali Zolghadr, Rebecca G. Ong, David R. Shonnard, Stephen M. Techtmann

**Affiliations:** a Department of Biological Sciences, Michigan Technological University, Houghton, Michigan, USA; b Department of Chemical Engineering, Michigan Technological University, Houghton, Michigan, USA; University of Minnesota Twin Cities

**Keywords:** environmental microbiology, biodegradation, pyrolysis, plastic chemical deconstruction, microbial ecology, plastic degradation metabolisms

## Abstract

Over half of the world’s plastic waste is landfilled, where it is estimated to take hundreds of years to degrade. Given the continued use and disposal of plastic products, it is vital that we develop fast and effective ways to utilize plastic waste. Here, we explore the potential of tandem chemical and biological processing to process various plastics quickly and effectively. Four samples of compost or sediment were used to set up enrichment cultures grown on mixtures of compounds, including disodium terephthalate and terephthalic acid (monomers of polyethylene terephthalate), compounds derived from the chemical deconstruction of polycarbonate, and pyrolysis oil derived from high-density polyethylene plastics. Established enrichment communities were also grown on individual substrates to investigate the substrate preferences of different taxa. Biomass harvested from the cultures was characterized using 16S rRNA gene amplicon sequencing and shotgun metagenomic sequencing. These data reveal low-diversity microbial communities structured by differences in culture inoculum, culture substrate source plastic type, and time. Microbial populations from the classes *Alphaproteobacteria*, *Gammaproteobacteria*, *Actinobacteria*, and *Acidobacteriae* were significantly enriched when grown on substrates derived from high-density polyethylene and polycarbonate. The metagenomic data contain abundant aromatic and aliphatic hydrocarbon degradation genes relevant to the biodegradation of deconstructed plastic substrates used here. We show that microbial populations from diverse environments are capable of growth on substrates derived from the chemical deconstruction or pyrolysis of multiple plastic types and that paired chemical and biological processing of plastics should be further developed for industrial applications to manage plastic waste.

**IMPORTANCE** The durability and impermeable nature of plastics have made them a popular material for numerous applications, but these same qualities make plastics difficult to dispose of, resulting in massive amounts of accumulated plastic waste in landfills and the natural environment. Since plastic use and disposal are projected to increase in the future, novel methods to effectively break down and dispose of current and future plastic waste are desperately needed. We show that the products of chemical deconstruction or pyrolysis of plastic can successfully sustain the growth of low-diversity microbial communities. These communities were enriched from multiple environmental sources and are capable of degrading complex xenobiotic carbon compounds. This study demonstrates that tandem chemical and biological processing can be used to degrade multiple types of plastics over a relatively short period of time and may be a future avenue for the mitigation of rapidly accumulating plastic waste.

## INTRODUCTION

Since the large-scale production of plastics began in the 1950s, plastic products have been a daily staple for many people around the world (1, 2). The durable and versatile nature of synthetic plastics makes them excellent choices for packaging, medical, and construction applications (2) and has resulted in the mass production of plastic, especially for single-use applications (1, 3). The durable qualities of plastics that make them so popular for various uses are their major downfall, especially when it comes to the disposal and degradation of these products (1, 2). Recent estimates indicate that ~60 to 70% of plastics are discarded and accumulating in landfills and the natural environment (1, 2, 4, 5). Under natural conditions in marine and terrestrial environments, plastic waste undergoes physical weathering that generates small plastic fragments and microplastics (6). Half-life degradation rates of plastic under abiotic conditions range from 10s to 1,000s of years (2). Slow degradation rates are concerning, especially since plastic production and use rates are expected to increase globally (7), which will ultimately result in more landfilled plastic waste under current disposal practices. The accumulation of plastic waste and microplastics in marine and terrestrial environments is concerning (3, 4, 6) because the negative impacts of waste on biogeochemical cycles, wildlife, and human health are not fully known (4, 6).

Since natural attenuation is not an efficient way to deal with solid plastic waste, chemical and biological methods have been investigated to increase the speed with which plastics can be broken down (5, 8–17). Since plastic depolymerization is often the slowest and most biologically challenging step of plastic degradation (2, 4, 16), many chemical and biological methods have focused on speeding up this part of the process (9–13, 16, 18). Products derived from depolymerization can be used to regenerate polymers or create other valuable products (10, 11, 13, 15, 16, 19). Additionally, depolymerization products are often more bioavailable and can be used as feedstocks for further biological processing of plastic wastes (5, 9, 16, 17, 20–23). Popular methods for the chemical deconstruction of plastics include thermal depolymerization (i.e., using heat to depolymerize plastics) and solvolysis (i.e., using solvents to depolymerize plastics) (10, 16, 19). Thermal depolymerization (e.g., pyrolysis) is an especially promising method for the deconstruction of plastics with C-C backbones, such as polypropylene (PP), low-density polyethylene (LDPE), and high-density polyethylene (HDPE) (9, 10, 13, 18, 19), although pyrolysis operations need to be finely tuned in order to consistently produce the desired products, which can be challenging to do if feedstock composition varies (13, 16, 24). Solvolysis (e.g., hydrolysis, aminolysis, alcoholysis, and glycolysis) is a promising method for the deconstruction of plastics with more labile chemical linkages (e.g., esters and urethanes), such as polyethylene terephthalate (PET) and polycarbonate (PC) (10, 11, 15, 19, 25, 26). Microorganisms have been shown to degrade plastics, including HDPE, LDPE, PP, PET, PC, polystyrene (PS), polyvinyl chloride, and polyurethane (PU) (4, 8, 12, 14, 16, 27–32), although the degradation and depolymerization of solid plastics are slow and are often the rate-limiting steps (2, 8, 12, 14).

In contrast, microorganisms show potential for the efficient degradation of products that are produced by the depolymerization of plastics using other processes (5, 16, 17, 33–38). This degradation improves on chemical deconstruction where it is more challenging to selectively generate value-added products (13, 16, 24), but products obtained from the chemical deconstruction of plastic have been successfully degraded by microbial isolates or communities into neutral (e.g., carbon dioxide or biomass) (17) or commercially valuable products [e.g., polyhydroxyalkanoate, poly(amide urethane), gallic acid, pyrogallol, catechol, muconic acid, vanillic acid, glycolic acid, and rhamnolipids] (33, 35–37). The conversion of plastic depolymerization products into neutral or value-added products has been demonstrated with PS (33), PET (35, 37), HDPE (17), and PU (36) over the course of hours or days. Paired chemical and biological processing of plastic wastes can substantially decrease the time needed to degrade plastics and could be scaled for future industrial use and product development (5, 16, 34, 38). The continued optimization of enzymatic function (12, 39) and microbial community function (in cases where isolates are not used) are current and future avenues of investigation that will make paired chemical and biological processing of plastic waste possible for more plastic types and on shorter time scales (5, 16, 34). As these tandem systems are developed, it will be important to perform technoeconomic and life cycle assessment analyses and compare them with other chemical, mechanical, and biological methods that are being developed to ensure that the most efficient process is selected and developed for use at a global scale (16, 38). Most previous work on the coupled chemical and biological processing of plastics has focused on isolates or communities using one plastic type (17, 23, 33, 35–37, 40, 41), although there is interest in using microbial communities to degrade mixed plastic waste following enzymatic depolymerization (5, 17, 42).

In this study, we explore the capacity of microbial communities to grow on individual and mixed depolymerization products derived from the chemical deconstruction of PET and PC and pyrolysis of HDPE as their sole carbon source. We enriched microbial communities capable of growth on these substrates from four diverse compost and sediment inocula and investigated patterns in the taxonomic and microbial community diversity of culture communities when grown on different plastic derivatives. Additionally, we utilized shotgun metagenomic sequencing to investigate the metabolic capacity of enriched organisms to break down the provided plastic-derived substrates. Our data show that low-diversity microbial communities dominated by members from the *Rhizobiales*, *Bacillales*, and *Burkholderiales* orders are enriched when grown on plastic derivative substrates. Microbial populations (defined here as “a group of organisms of the same species” [43]) from the classes *Alphaproteobacteria* and *Gammaproteobacteria* were observed to be significantly enriched when grown on substrates derived from HDPE, and microbial populations from the classes *Actinobacteria*, *Acidobacteriae*, *Alphaproteobacteria*, and *Gammaproteobacteria* were observed to be significantly enriched when grown on substrates derived from PC. These communities contain abundant aromatic and aliphatic hydrocarbon degradation genes, which likely allows for their sustained growth on these complex anthropogenic waste compounds.

## RESULTS

### Taxonomic diversity of enrichment and single-substrate cultures.

Four different compost or sediment samples (iron-rich stream sediment, Lake Superior sediment, vermicompost, and Caspian Sea sediment) were used to inoculate aqueous enrichment cultures in minimal medium with depolymerized PC and products expected from the hydrolysis or aminolysis of PET and pyrolysis of HDPE plastics as the sole carbon sources. Cultures were grown on various combinations of disodium terephthalate (derivative from PET hydrolysis); terephthalamide (derivative from PET aminolysis); chemically deconstructed PC (hereinafter referred to as bisphenol A [BPA]); a 1:1:1:1 mixture of hexene, decene, hexadecene, and eicosene (hereinafter referred to as alkene mixture; HDPE derivatives); and pyrolyzed HDPE (HDPE derivatives) as described below. DNA was extracted from microbial communities harvested from aqueous culture as described below. The following assessments of diversity and microbial populations enriched under different conditions are based on a data set generated from gene amplicon sequencing of the V4 region of the 16S rRNA gene. It is important to note that gene amplicon sequencing does not provide an absolute quantification of microorganisms within samples. The results of this work have been interpreted with this technical limitation in mind. The data set used in this study consists of 70 samples, 1,044,047 reads, and 5,877 operational taxonomic units (OTUs) clustered at a 3% distance threshold as described below. The count and taxonomy tables used for the following analyses are published online at FigShare (https://figshare.com/projects/Microbial_Deconstructed_Plastic_Substrate_Preferences/131882).

The initial characterization of the data set revealed that many samples sequenced poorly, retaining only 10s to 100s of reads. Because trends in community diversity between samples cannot be assessed accurately in samples with so few reads, a rarefaction curve was used to determine the minimum sequencing depth required to adequately represent samples within this data set. A rarefaction curve (see Fig. S1 in the supplemental material) revealed that samples with 3,000 or more reads have been sufficiently sequenced and are representative of sample identity and biodiversity. Based on this result, samples with fewer than 3,000 reads were removed from the data set prior to further analyses. The taxonomic diversity of samples discarded from the data set is explored within the Appendix in the supplemental material.

**FIG 1:**
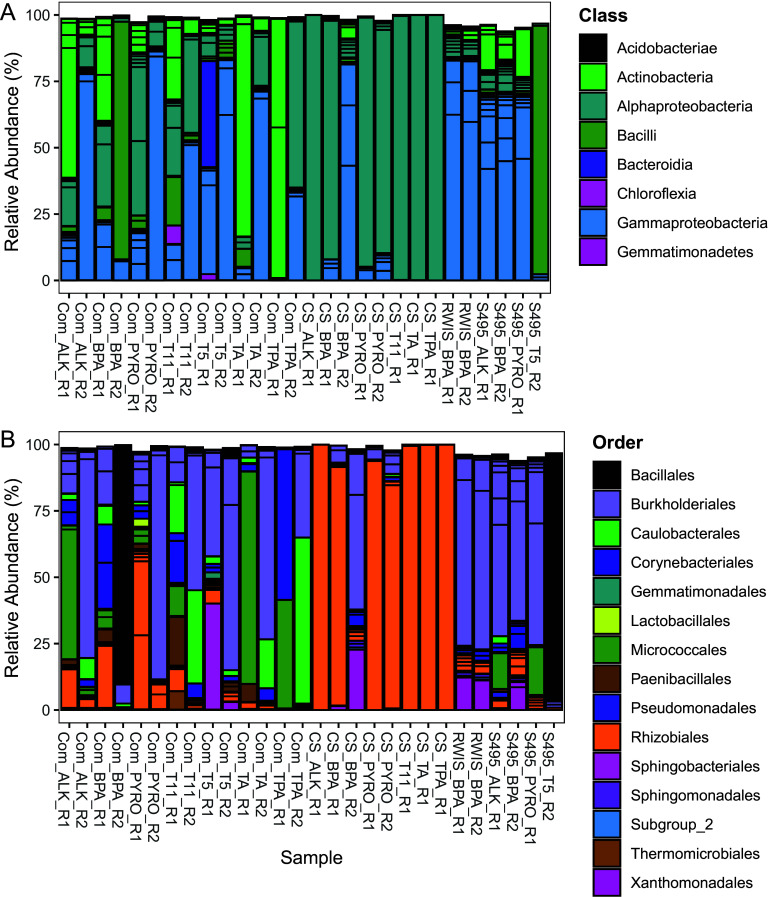
Stacked bar plots showing the relative abundance of the 50 most abundant OTUs within each sample by taxonomic class (A) and order (B). The 50 most abundant OTUs in the data set were determined by assessment of the normalized abundance of OTUs in the 16S rRNA gene amplicon data set. For cultures grown on mixed substrates, sample names used the following convention: “Incolum Source_Transfer Number_Biological Replicate.” For cultures grown on individual substrates sample names used the following convention: “Inoculum Source_Substrate_Biological Replicate.” COM, vermicompost; CS, Caspian Sea sediment; RWIS, iron-rich stream sediment; S495, Lake Superior sediment; T5, transfer 5 (10 weeks time); T11, transfer 11 (22 weeks time); R1, biological replicate 1; R2, biological replicate 2; ALK, alkene mixture; BPA, deconstructed PC; PYRO, pyrolysis; TPA, terephthalamide; TA, disodium terephthalate; CONT, biological control (no carbon substrate provided); B, blank (biology-free medium and substrate blanks).

Within retained samples, the taxonomy of the 50 most abundant OTUs (identified using the normalized abundance of OTUs within each sample) in the data set was assessed in detail. These organisms account for 98.35% of community composition on average (Table S1 in the supplemental material is available online at https://doi.org/10.6084/m9.figshare.19126115). Based on the dominance of the 50 OTUs, an analysis of these OTUs will provide a comprehensive assessment of the taxonomic diversity contained within culture communities. Culture communities are dominated by organisms from the classes *Alphaproteobacteria* (0.26 to 99.93%, 36.65% average) and *Gammaproteobacteria* (0.03 to 87.91%, 36.51% average) (Fig. 1A; Table S1 available online at https://doi.org/10.6084/m9.figshare.19126115). *Alphaproteobacteria* are especially dominant with cultures originally inoculated with sediment from the Caspian Sea (7.17 to 99.93%, 84.88% average), *Actinobacteria* are abundant within many of the cultures originally inoculated with vermicompost (0.34 to 97.95%, 24.89% average), and *Bacilli* are dominant within a few samples derived from various inocula (0 to 93.55%, 8.55% average) (Fig. 1A; Table S1 available online at https://doi.org/10.6084/m9.figshare.19126115). Dominant taxonomic orders include *Rhizobiales* (0.10 to 99.93%, 30.30% average), *Bacillales* (0 to 89.46%, 7.09% average), and *Burkholderiales* (0.04 to 87.87%, 34.07% average) (Fig. 1B; Table S1 available online at https://doi.org/10.6084/m9.figshare.19126115). Rhizobiales are dominant in cultures originally inoculated with sediment from the Caspian Sea (4.64 to 99.93%, 84.38% average), while *Bacillales* (0 to 89.46%, 7.09% average) and *Burkholderiales* (0.04 to 87.87%, 34.07% average) are found within the majority of the cultures, regardless of the initial soil inoculum (Fig. 1B; Table S1 available online at https://doi.org/10.6084/m9.figshare.19126115). Organisms from the order *Paenibacillales* are prominent within a number of the cultures originally inoculated with vermicompost (0.21 to 18.71%, 3.01% average) (Fig. 1B; Table S1 available online at https://doi.org/10.6084/m9.figshare.19126115). *Xanthomonadales* are prevalent in a few cultures that were grown on BPA (0.01 to 22.72%, 8.34% average) (Fig. 1B; Table S1 available online at https://doi.org/10.6084/m9.figshare.19126115). In general, cultures originally inoculated with vermicompost display greater diversity than those inoculated with the other sediments (Caspian Sea sediment, Lake Superior sediment, and iron-rich stream sediment) (Fig. 1A and B).

### Microbial community diversity patterns.

**FIG 2:**
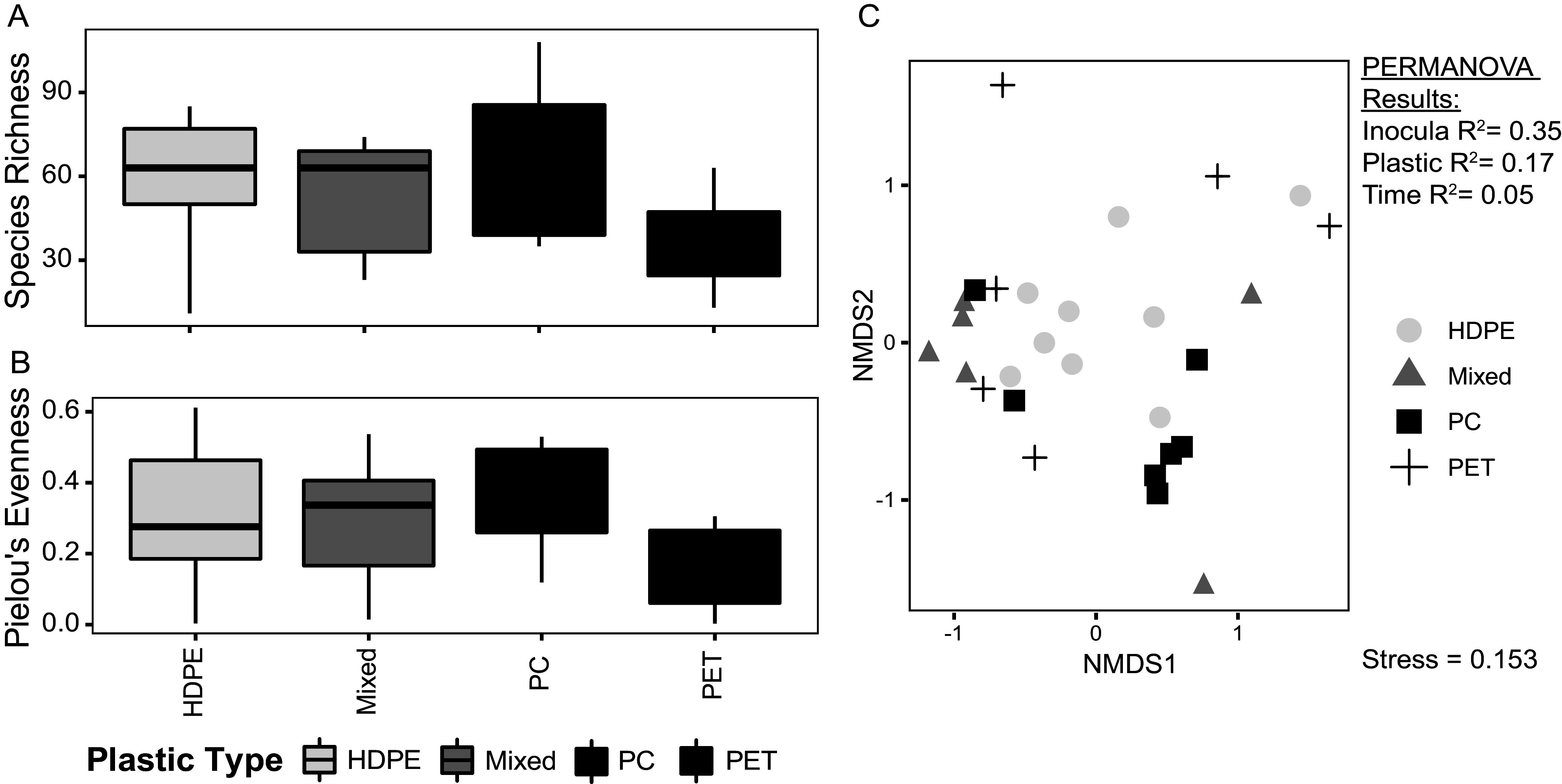
Boxplots showing richness (A) and Pielou’s evenness index (B) for microbial communities grown on different plastic derivative substrates. Boxplots (A and B) display the distribution of data within each category, where the main colored box displays the interquartile range (25th to 75th percentile) of the data. Solid lines within the boxes represent the median of the data. Whiskers on the plot display the maximum and minimum expected values of the data distribution. The nonmetric multidimensional scaling (NMDS) plot show the microbial community 16S rRNA gene relative abundances using a Bray-Curtis dissimilarity (C). R^2^ values for environmental variables that were significantly correlated with changes in community composition (*P* ≤ 0.05) in a PERMANOVA analysis (Table S2) are listed on the plot. Points on the NMDS plot (C) represent individual microbial community samples and are colored based on the substrate plastic type with which each sample was cultured. Points that lie close together are more similar in community composition, while points that lie far apart are less similar in community composition. HPDE, high-density polyethylene; PC, polycarbonate; PET, polyethylene terephthalate; mixed, HDPE, PC, and PET derivative substrates mixed together.

Patterns in species richness (number of unique OTUs in a community) and evenness (Pielou’s evenness, how close in numerical abundance OTUs in a community are) of culture microbial communities were compared based on the plastic from which different substrates were derived. For example, both terephthalate and terephthalamide are derived from PET and thus would be grouped into a single category. Similarly, both the alkene mixture and the pyrolysis product are derived from polyolefin plastics, such as HDPE, and are thus grouped. Our cultured microbial communities displayed low diversity, containing on average 54 unique OTUs (Fig. 2A), and low evenness (mean Pielou’s evenness, 0.29). indicating that a few highly abundant taxa dominate community composition and the remaining microbial community members persist at low abundances (Fig. 2B). These results agree with observations of taxonomic diversity (Fig. 1A and B). Species richness and evenness do not differ greatly between cultures grown on mixed plastic type substrates, PC derivative substrates, and HDPE derivative substrates (Fig. 2A and B). Cultures grown on PET derivative substrates display lower richness and evenness than the other cultures investigated here (Fig. 2A and B).

A permutational multivariate analysis of variance (PERMANOVA) was performed on Bray-Curtis dissimilarities and associated culture metadata to determine how much variance in microbial community composition can be explained by differences in culturing conditions. This analysis shows that culture inoculum (R^2^ = 0.35), culture substrate plastic type (R^2^ = 0.17), and time (number of transfers) (R^2^ = 0.05) are significant primary drivers of microbial community dissimilarity within cultures (see Table S2 in the supplemental material). A large amount of variance (residuals R^2^ = 0.44) is unexplained (Table S2). When Bray-Curtis dissimilarities are visualized using a nonmetric multidimensional scaling (NMDS) plot, samples cluster based on culture substrate plastic type to some extent (Fig. 2C). Cultures grown on HDPE derivative substrates lie within the center of the plot, while cultures grown on mixed plastic type substrates and PC and PET derivative substrates lie around the perimeter of the plot (Fig. 2C). In general, culture microbial communities are highly dissimilar in terms of community composition (mean Bray-Curtis dissimilarity = 0.84), which is interesting given that samples within the data set are composed primarily of a small number of common OTUs (Table S1 available online at https://doi.org/10.6084/m9.figshare.19126115).

### Substrate preferences of microbial populations.

Assessments of microbial community diversity reveal that culture substrate (plastic type) is a significant driver of variation in microbial community composition (Table S2). While the starting inoculum of cultures drives more variation in culture community composition, the same dominant taxa are observed within many cultures, despite different starting inoculum sources (Table S1 available online at https://doi.org/10.6084/m9.figshare.19126115). The inocula used for these cultures came from diverse environments with very different environmental conditions. Based on this information, our PERMANOVA result (Table S2) indicating that the starting inoculum is a major factor driving microbial community dissimilarity is not surprising. We would expect different environments to host distinct microbial communities. Since the goal of this work is to investigate microbial community members capable of degrading different types of deconstructed plastic substrates, we further explored the role that culture substrate (plastic type) plays in driving community dissimilarity between cultures. We looked at differences in the relative abundance (identified using the normalized abundance of OTUs within each sample) of dominant microbial populations (Table S1 available online at https://doi.org/10.6084/m9.figshare.19126115) when grown on individual substrates derived from HDPE, PC, or PET to determine if any microbial taxa were significantly enriched when grown on one different plastic type derivative compared with the others. While quantitative substrate degradation rates would be ideal to pair with these data, analytical characterization and quantification of PET and PC derivatives (44–48) are technically challenging and were unfeasible to undertake for the scope of this work. Results and interpretations of the data are discussed with this technical limitation in mind.

**FIG 3:**
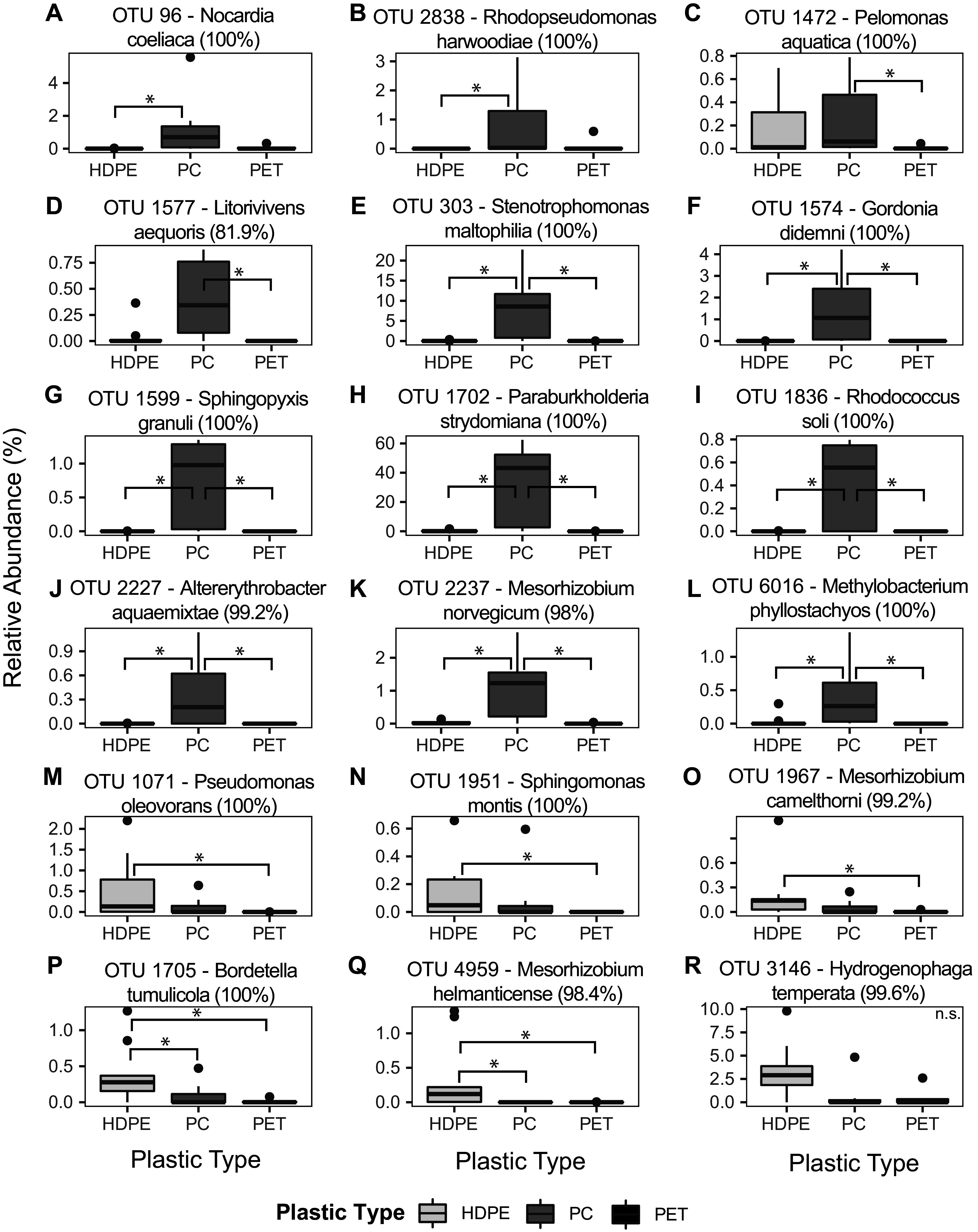
Boxplots showing the relative abundance (%) of OTU 96 (A), OTU 2838 (B), OTU 1472 (C), OTU 1577 (D), OTU 303 (E), OTU 1574 (F), OTU 1599 (G), OTU 1702 (H), OTU 1836 (I), OTU 2227 (J), OTU 2237 (K), OTU 6016 (L), OTU 1071 (M), OTU 1951 (N), OTU 1967 (O), OTU 1705 (P), OTU 4959 (Q), and OTU 3146 (R) in samples collected from cultures grown on substrates derived from HDPE, PET, or PC. Boxplots display OTUs with significant Kruskal-Wallis tests (*P* ≤ 0.05) (Table S3). Brackets with stars centered above them in the plots indicate the results of significant *post hoc* Dunn tests (*P* ≤ 0.025) (Table S4) and show culture-substrate condition pairs where OTU abundance is significantly different between two sets of culture conditions. Species taxonomy and percent identity as classified by MegaBLAST are displayed alongside each OTU ID. Boxplots display the distribution of data within each treatment, where the main-colored box displays the interquartile range (25th to 75th percentile) of the data. Solid lines within the boxes represent the median of the data. Whiskers on the plot display the maximum and minimum expected values of the data distribution, and black-colored points represent outlier data points with respect to the plotted distribution. HDPE, high-density polyethylene; PC, polycarbonate; PET, polyethylene terephthalate; n.s., nonsignificant Dunn test.

Kruskal-Wallis tests (see Table S3 in the supplemental material) and *post hoc* Dunn tests (see Table S4 in the supplemental material) were used to identify OTUs observed at significantly greater abundances when grown on individual substrates derived from one of the investigated plastic types (HDPE, PET, and PC) compared with the others. Only the 50 most abundant OTUs in the data set were assessed here due to their dominance over community composition (Table S1 available online at https://doi.org/10.6084/m9.figshare.19126115), as described above. This analysis identified 12 of the top 50 OTUs that were significantly more abundant in cultures grown on PC derivative substrates (Fig. 3A to L) and six OTUs that were significantly more abundant in cultures grown on HDPE derivative substrates (Fig. 3M to R).

OTU 96 and OTU 2838 were enriched when grown on PC-derived substrates compared with HDPE-derived substrates (Fig. 3A and B). OTU 1472 and OTU 1577 were enriched when grown on PC-derived substrates compared with PET-derived substrates (Fig. 3C and D). OTU 303, OTU 1574, OTU 1599, OTU 1702, OTU 1836, OTU 2227, OTU 2237, and OTU 6016 were enriched when grown on PC-derived substrates compared with HDPE and PET-derived substrates (Fig. 3E to L). Generally, these OTUs comprise ~1 to 5% relative abundance within communities grown on PC-derived substrates (Fig. 3A to D, F, G, and I to L), while OTUs 303 and 1702 comprise 10% and 40% of community composition on average when grown on PC derived substrates, respectively (Fig. 3E and H).

OTU 1071, OTU 1951, and OTU 1967 were enriched when grown on HDPE-derived substrates compared with PET-derived substrates (Fig. 3M to O). OTU 1705 and OTU 4959 were enriched when grown on HDPE-derived substrates compared with PC- and PET-derived substrates (Fig. 3P and Q). OTU 3146 was identified as having a significantly different mean abundance compared between groups by a Kruskal-Wallis test (Table S3) but did not have a significant *post hoc* Dunn test result (Table S4). OTU 3146 appears to be more abundant in communities grown on HDPE-derived substrates than communities grown on PC- and PET-derived substrates (Fig. 3R). Higher relative abundance values visualized as outlier points in both the PC and PET box plots may have affected the significance of the *post hoc* Dunn test for OTU 3146 (Fig. 3R). Generally, these OTUs comprise ~1% relative abundance within communities grown on HDPE-derived substrates (Fig. 3M to Q), while OTU 3146 comprises ~2.5% relative abundance on average within communities grown on HDPE-derived substrates (Fig. 3R).

### Metabolic pathways relevant to plastic degradation.

During the enrichment process, the second biological replicate of the vermicompost enrichment culture (see 16S rRNA samples Com_T5_R2 and Com_T11_R2) consistently grew well when grown on the mixed substrates. Given the observed potential of this culture to grow on deconstructed plastic substrates, shotgun metagenomic sequencing was performed on DNA extracted from culture biomass after 10 weeks of growth (see 16S sample Com_T5_R2). Metagenomic data obtained from this sample are used to explore the metabolic pathways that microbial populations within these enrichment cultures may be using to degrade the deconstructed plastic substrates provided to them. While these data do not include metagenomic information from cultures enriched from the other three starting soils (iron-rich stream sediment, Lake Superior sediment, and Caspian Sea sediment), we feel they provide useful information that can guide future functional assessments of metabolic processes used to degrade deconstructed plastic substrates, especially given the high taxonomic overlap observed within these cultures (Table S1 available online at https://doi.org/10.6084/m9.figshare.19126115). The following assessments of metabolic potential in deconstructed plastic-degrading cultures are derived from the analysis of a single metagenomic sample (see 16S rRNA sample Com_T5_R2). We assess genes and metabolic pathways identified in the bulk assembled metagenomic contigs and in high-quality metagenome assembled genomes (MAGs) binned from these data (see Materials and Methods).

**FIG 4:**
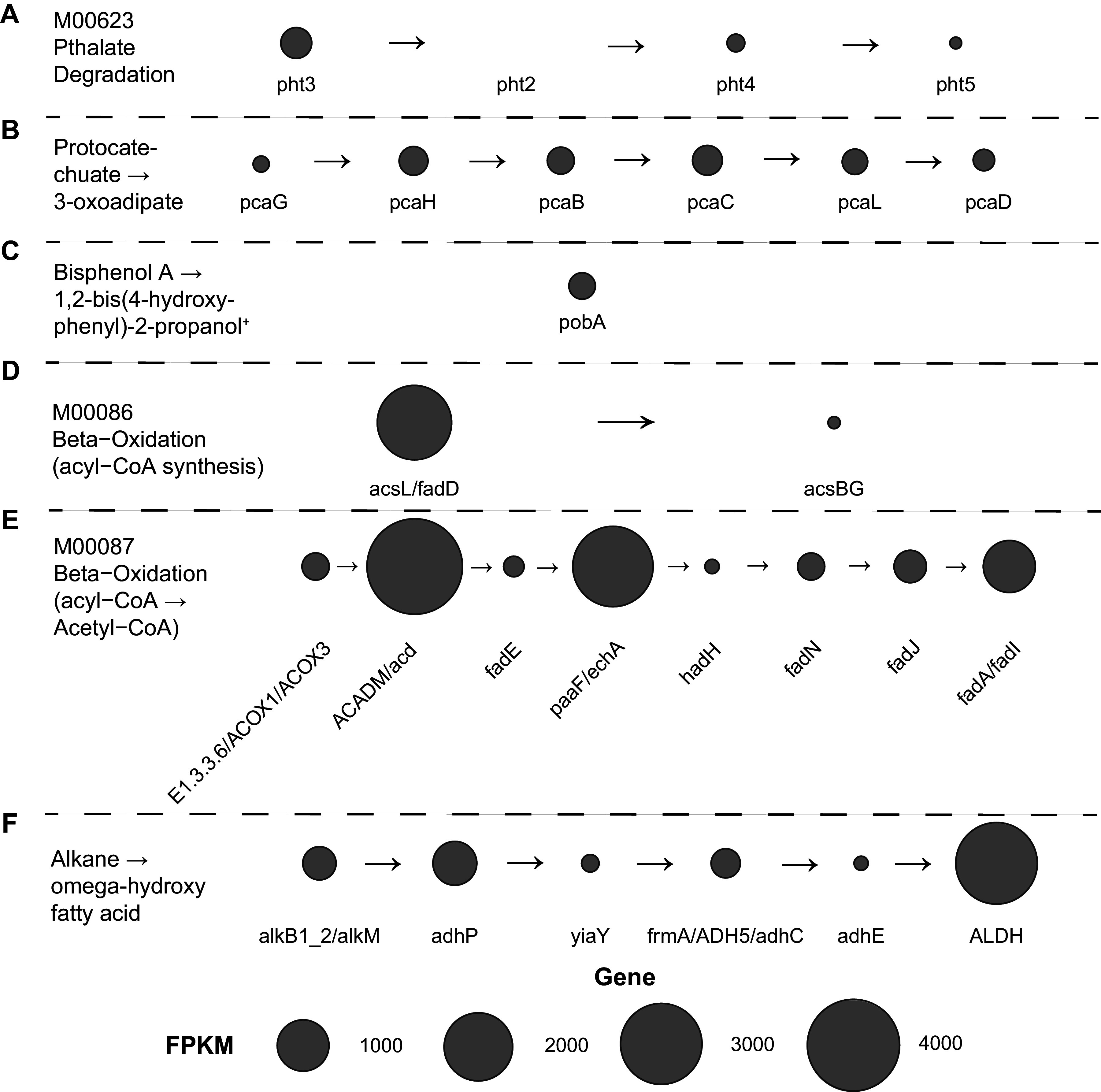
Bubble plot displaying the summed gene abundance of genes from assembled metagenomic contigs within the KEGG phthalate degradation module (A), protocatechuate to 3-oxoadipate degradation pathway (B), beta-oxidation (acyl-CoA synthesis) module (C), beta-oxidation (acyl-CoA to Acetyl-CoA) module (D), and alkane to omega-hydroxy fatty acid degradation pathway (E). Bubble size corresponds with the detected abundance of each gene, where larger bubbles represent genes detected at high abundances and smaller bubbles represent genes detected at low abundances. Gene abundances are plotted as metagenome fragments per kilobase of predicted protein sequence per million mapped reads (FPKM). +, Bisphenol A degradation to 1,2-bis(4-hydroxyphenyl)-2-propanol by the *pobA* enzyme proposed by Zhou and colleagues (51). Genes in each pathway are assembled in order of use in substrate degradation from left to right with arrows in between genes representing the direction of gene use in the pathway.

A brief taxonomic assessment of the metagenomic contigs can be found in the Appendix. Annotated functional protein sequences from metagenomic contigs were analyzed using GhostKOALA (49) to identify metabolic pathways of interest that microbial populations in these cultures may utilize to break down aromatic (PET- and PC-derived substrates) and aliphatic (HDPE-derived substrates) hydrocarbon substrates. This analysis identified 12 Kyoto Encyclopedia of Genes and Genomes (KEGG) modules/pathways relevant to the degradation of aromatic compounds (Fig. 4A to C; see Fig. S2A to J in the supplemental material) and three KEGG modules/pathways relevant to the degradation of aliphatic hydrocarbon compounds (Fig. 4D to F). Terephthalate and other phthalate compounds are often degraded to protocatechuate and are subsequently converted to pyruvate which can be used in multiple downstream metabolic processes (50). A near-complete phthalate degradation pathway (missing phthalate 4,5-dioxygenase reductase component [*pht2*]) was identified within the metagenomic data as well as the complete pathway for the degradation of protocatechuate (Fig. 4A and B). While the pathway and enzymes involved in the degradation of BPA are not completely known, the initial step of degradation appears to be catalyzed by cytochrome P450 or *p*-hydroxybenzoate hydroxylase (*pobA*) (51, 52). Genes encoding *pobA* were detected within the metagenomic data (Fig. 4C). These data indicate that microbial populations within the sample are likely capable of degrading terephthalate and other phthalate compounds and may have the potential to catalyze the initial step of BPA degradation (51). In addition to the genes and pathways relevant to the degradation of terephthalate, phthalates, and BPA, a number of other aromatic hydrocarbon metabolisms that could be relevant to plastic degradation were identified (53, 54). Descriptions of these pathways can be found in the Appendix and visualized in Fig. S2.

In addition to the promising capacity to degrade aromatic compounds, the metagenomic data revealed complete pathways for the degradation of fatty acids and aliphatic hydrocarbons (Fig. 4D to F). The complete KEGG modules for the β-oxidation of hexadecanoic acid to palmitoyl-coenzyme A (CoA) (Fig. 4D) and β-oxidation of various acyl-CoA compounds to acetyl-CoA (Fig. 4E) were identified in the metagenomic data. β-Oxidation has been identified previously as an important process in the degradation of *n*-alkanes present in marine oil spills (55). Additionally, the complete pathway for alkane degradation to ω-hydroxy fatty acids utilizing alkane 1-monooxygenase (*alkB*) and various alcohol/aldehyde dehydrogenases (*adhP*, *yiaY*, *frm*A, *adhE*, and *ALDH*) was identified in the metagenomic data (Fig. 4F), which is a well-known pathway of alkane degradation (56, 57).

**FIG 5:**
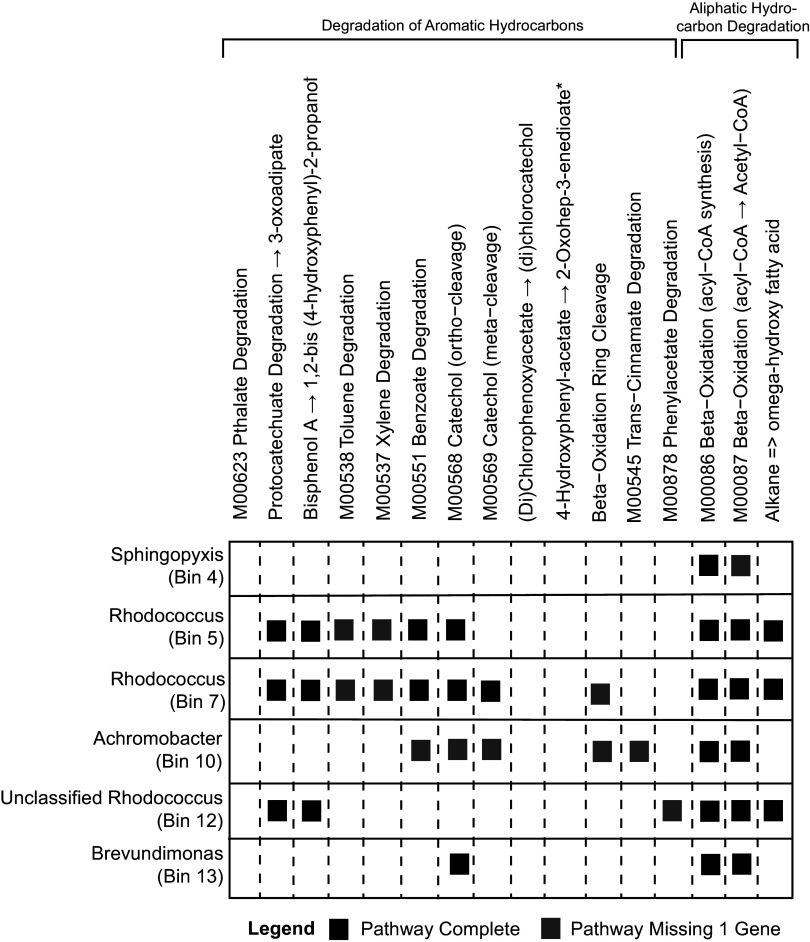
Chart displaying the presence of complete or near-complete (missing 1 gene) KEGG modules and pathways relevant to the degradation of aromatic and aliphatic hydrocarbons within high-quality MAGs. +, Bisphenol A degradation to 1,2-bis(4-hydroxyphenyl)-2-propanol by the *pobA* enzyme proposed by Zhou and colleagues (51). *, Degradation of 4-hydroxyphenyl-acetate occurs via the homoprotocatechuate degradation pathway (KEGG module M0053; *hpaD/hpcB*, *hpaE/hpcC*, *hpaF*/*hpcD*, and *hpaG* genes).

Binning of metagenomic data resulted in the recovery of six high-quality and nearly complete MAGs. Three of the MAGs were classified in the *Rhodococcus* genus while the other three MAGs were classified in the *Sphingopyxis*, *Achromobacter*, and *Brevundimonas* genera. Each MAG was assessed for the presence of the KEGG modules and pathways found in the bulk metagenomic data (Fig. 4). The *Sphingopyxis* bin (bin 4) contains the complete pathway for β-oxidation of hexadecanoic acid to palmitoyl-CoA and a nearly complete pathway for β-oxidation of various acyl-CoA compounds to acetyl-CoA (Fig. 5). The first *Rhodococcus* bin (bin 5) contains complete pathways for the degradation of protocatechuate to 3-oxoadipate, benzoate degradation, catechol degradation (*ortho*-cleavage), β-oxidation of hexadecanoic acid to palmitoyl-CoA, β-oxidation of various acyl-CoA compounds to acetyl-CoA, and the degradation of alkanes to ω-hydroxy fatty acids (Fig. 5). Additionally, *Rhodococcus* bin 5 contains near-complete pathways for the degradation of toluene and xylene (Fig. 5). The second *Rhodococcus* bin (bin 7) contains the same complete and near-complete pathways as those observed in *Rhodococcus* bin 5 and also contains the complete pathway for the degradation of catechol using *meta*-cleavage and a near-complete pathway for β-oxidation ring cleavage (Fig. 5). The *Achromobacter* bin (bin 10) contains complete pathways for β-oxidation of hexadecanoic acid to palmitoyl-CoA and β-oxidation of various acyl-CoA compounds to acetyl-CoA and contains near-complete pathways for benzoate degradation, catechol degradation (*ortho*- and *meta*-cleavage), β-oxidation ring cleavage, and *trans*-cinnamate degradation (Fig. 5). The third *Rhodoccocus* bin (bin 12) contains complete pathways for the degradation of protocatechuate to 3-oxoadipate, β-oxidation of hexadecanoic acid to palmitoyl-CoA, β-oxidation of various acyl-CoA compounds to acetyl-CoA, and the degradation of alkanes to ω-hydroxy fatty acids and a near-complete pathway for phenylacetate degradation (Fig. 5). The *Brevundimonas* bin (bin 13) contains complete pathways for β-oxidation of hexadecanoic acid to palmitoyl-CoA, β-oxidation of various acyl-CoA compounds to acetyl-CoA, and catechol degradation (*ortho*-cleavage) (Fig. 5). Interestingly, complete or near-complete pathways for phthalate degradation, the degradation of (di)chlorophenoxyacetate to (di)chlorocatechol, and the degradation of 4-hydroxyphenyl-acetate to 2-oxohep-3-enedioate were not observed in any of the six high-quality MAGs (Fig. 5).

Overall, the metagenomic data reveal multiple pathways with abundant genes for the complete degradation of aromatic and aliphatic hydrocarbons relevant to the biodegradation of deconstructed plastic substrates utilized in these experiments. This information can be used to guide future functional assessments of the activity of genes in these pathways during growth on deconstructed plastic substrates. Files from Prokka, count data from HTSeq, and KEGG ortholog values obtained from GhostKOALA used to explore metabolic pathways in KEGG and generate Fig. 4 and 5 and Fig. S6 in the supplemental material can be found online at FigShare (https://figshare.com/projects/Microbial_Deconstructed_Plastic_Substrate_Preferences/131882).

## DISCUSSION

Gene amplicon and shotgun metagenomic sequencing of cultured microbial communities grown on products from the chemical deconstruction of PET and PC and pyrolysis of HDPE reveal low-diversity microbial communities (Fig. 2A) dominated by members of the *Alphaproteobacteria* and *Gammaproteobacteria* classes (Fig. 1A). Differences in substrate composition and initial inoculum source were found to be primary drivers of variation in microbial community composition (Fig. 2C). Twelve OTUs were found to be significantly more abundant when grown on PC-derived substrates alone (Fig. 3A to L), of which two accounted for large proportions of the total community composition (Fig. 3E and H). Six OTUs were found to be significantly more abundant when grown on HDPE-derived substrates alone (Fig. 3M to R). An analysis of bulk metagenomic data and high-quality MAGs reveal multiple pathways for the degradation of aromatic and aliphatic hydrocarbon compounds (Fig. 4 and 5). Here, we discuss factors that may drive differences in culture community composition over time, the capacity of OTUs identified as significantly more abundant when grown on PC or HDPE (Fig. 3) to use the respective substrate based on information in the literature, and the role of metabolic pathways identified in the metagenomic data (Fig. 4; Fig. S2) and MAGs (Fig. 5) in plastic biodegradation.

Assessments of microbial community taxonomy and diversity revealed that the 50 most abundant OTUs account for 98.35% of community composition on average (Table S1 available online at https://doi.org/10.6084/m9.figshare.19126115) and that microbial communities are composed of 54 OTUs on average and are dominated by a few highly abundant taxa (mean Pielou’s evenness, 0.29) (Fig. 2A and B). This finding is in line with taxonomic observations of the 50 most abundant OTUs where organisms from the taxonomic orders *Rhizobiales* (30.30% average), *Bacillales* (7.09% average), and *Burkholderiales* (34.07% average) comprise 71.46% of the culture community composition on average (Fig. 1B; Table S1 available online at https://doi.org/10.6084/m9.figshare.19126115). These taxonomy and alpha diversity measures agree that a small group of highly abundant organisms dominates microbial community composition within these cultures. Some of the samples in the data set sequenced poorly and were discarded from the data set for further analyses. Potential reasons for poor sequencing of certain culture samples are further explored in the Appendix. Despite the low observed diversity within individual cultures, beta diversity measures indicate that culture microbial communities are highly dissimilar from each other (mean Bray-Curtis, 0.84) and that differences in community composition are driven primarily by differences in culture inoculum (R^2^ = 0.35), culture substrate plastic type (R^2^ = 0.17), and time (R^2^ = 0.05) (Fig. 2C; Table S2). The Bray-Curtis dissimilarity measure calculates the sample-to-sample dissimilarity of each species weighted by its total relative abundance in the pair of samples being assessed (58). Therefore, abundant species are more heavily weighted in the calculation than rare species (58), and the variation in the abundance of abundant species between samples can more heavily influence the calculation. Although a small number of OTUs dominate the community composition of all cultures (Table S1 available online at https://doi.org/10.6084/m9.figshare.19126115), variation in the abundance of dominant taxa between cultures and differences in the taxonomy and abundance of rare community members are likely the primary drivers behind the high observed dissimilarity between communities. Additionally, isolation of culture microbial communities from one another can enhance the influence of ecological drift (i.e., random changes in the relative abundance of microbial community members [43, 59]) over time, increasing microbial community dissimilarity, especially within low- diversity communities (43, 60–63). The role of ecological drift is further explored in the S.I. Appendix.

An analysis of the relative abundance of the top 50 OTUs in cultures grown on individual plastic derivative substrates revealed 12 OTUs that were significantly more abundant when grown on PC-derived substrates (Fig. 3A to L) and six OTUs that were significantly more abundant when grown on HDPE-derived substrates (Fig. 3M to R). Organisms identified as significantly more abundant when grown on HDPE-derived substrates have been shown previously to grow on aliphatic hydrocarbons (64–70). Pseudomonas oleovorans (Fig. 3M) has been shown to degrade alkenes and *n*-alkanes (64), and many Pseudomonas species are capable of degrading aliphatic hydrocarbons (68). Members of the *Sphingomonas* (Fig. 3N), *Mesorhizobium* (Fig. 3O and Q), and *Bordetella* (Fig. 3P) genera have also been shown to degrade aliphatic hydrocarbons (65–67, 69, 70). It is also important to note that members of the Pseudomonas, *Sphingomonas*, *Mesorhizobium*, and *Bordetella* genera have also been implicated in the degradation of aromatic compounds and polyesters (8, 14, 50, 71–73), including BPA (74, 75), which is discussed more below for organisms enriched during growth on deconstructed PC. Despite a high relative abundance in cultures with HDPE-derived substrates, we did not find evidence in the literature that organisms from the *Hydrogenophaga* genus (Fig. 3R) are capable of using aliphatic hydrocarbons, although there is good evidence that they are capable of utilizing polycyclic aromatic hydrocarbons (PAHs) (76, 77). OTU 3146, a close relative of Hydrogenophaga temperata (Fig. 3R), had a nonsignificant *post hoc* Dunn test result (Table S3 and S4), and thus, it was not significantly enriched in any culturing condition compared with another.

The literature supports that the organisms we found to be enriched when grown on PC-derived substrates are likely capable of using the substrates. Members of the *Rhodococcus* (Fig. 3I), *Methylobacterium* (Fig. 3L), and *Sphingopyxis* (Fig. 3G) genera have previously been implicated in the degradation of BPA (75, 78), which is expected to be one of the primary products of PC aminolysis or hydrolysis (26). Additionally, members of *Methylobacterium* have been shown to degrade other PAHs (79); members of *Sphingopyxis* have been shown to degrade styrene, phenols, and PAHs (80); and members of the *Rhodococcus* genus have also been shown to degrade phthalates, including terephthalate, as well as PS, PP, and polyethylene plastics (50, 73). Members of the *Stenotrophomonas* genus (Fig. 3E) have been shown to degrade environmental estrogens (81) and multiple oil-derived PAHs (82). Stenotrophomonas maltophilia (OTU 303) has been shown to grow on lignin (72). Interestingly, many lignin-degrading organisms are capable of degrading polyethylene plastics (8, 14, 73, 83). Stenotrophomonas maltophilia (OTU 303) (Fig. 3E) comprises approximately 10% of community composition in cultures grown with deconstructed PC and may play an important role in the degradation of PC-derived monomers, such as BPA. Paraburkholderia strydomiana (OTU 1702) (Fig. 3H) was also dominant within cultures grown with deconstructed PC (avg. 40% relative abundance). Members of the genus have been shown to degrade phenolic acids (84), indicating that this organism may play a role in BPA degradation. Members of the genus *Gordonia* (Fig. 3F) have been shown to degrade environmental estrogens, styrene, and phthalates (85–87). Members of the *Nocardia* genus (Fig. 3A) have been shown to degrade phthalates, PAHs, and a variety of other phenolic compounds and azo dyes (88–90). Members of the *Rhodopseudomonas* (Fig. 3B) and *Pelomonas* (Fig. 3C) genera have been shown to degrade various aromatic compounds (91–93), including polylactic acid by *Rhodopseudomonas* (94). Members of the *Altererythrobacter* (Fig. 3J) and *Mesorhizobium* (Fig. 3K) genera have been shown to degrade lignin (72, 95), which could give these organisms the capacity to degrade aromatic polymers, such as polyethylene and PC plastics, and their monomers (8, 14, 73, 83). We were not able to find any published evidence that members of the *Litorivivens* genus can degrade aromatic hydrocarbons, but many members of the *Gammaproteobacteria* class are capable of degrading aromatic compounds, including members of the *Paraburkholderia* and *Pelomonas* genera discussed here (84, 92, 93). These results indicate that many groups of microorganisms identified in the enriched communities have been implicated previously in the degradation of diverse plastic types.

In addition to the identification of numerous organisms that grow on PC- and HDPE-derived substrates (Fig. 3), an analysis of bulk metagenomic data and MAGs reveal diverse metabolic pathways involved in the degradation of aromatic and aliphatic hydrocarbons (Fig. 4 and 5; Fig. S2). Chemical depolymerization of PC and PET primarily produces BPA (PC), terephthalic acid (PET), and ethylene glycol (PET) which can be metabolized by microorganisms (26, 50, 74). Terephthalic acid (TPA) is degraded to protocatechuate using a dioxygenase (*tphA1A2A3*) to hydroxylate the TPA molecule in two locations followed by the use of a dehydrogenase (*tphB*) to remove a carboxyl group, producing protocatechuate (50), which can then be degraded to compounds, such as pyruvate and oxaloacetate (96), which can then be used for cellular respiration. The degradation pathway of ethylene glycol is also well known (50) but not discussed here since it was not a substrate used in our work. TPA dioxygenase (*tphA1A2A3*) and dehydrogenase (*tphB*) genes were not detected within our metagenomic data, although a near-complete phthalate degradation pathway (missing phthalate 4,5-dioxygenase reductase component [*pht2*]) was detected within the bulk metagenomic data (Fig. 4A). Additionally, the complete pathway for the degradation of protocatechuate to 3-oxoadipate was found in the bulk metagenomic data (Fig. 4B) and all three *Rhodococcus* MAGs (bins 5, 7, and 12) (Fig. 5). While a complete phthalate degradation pathway was not found in any of the high-quality MAGs (Fig. 5), the full pathway could be present within a lower quality MAG or phthalate degradation in the culture could be occurring through cross-feeding between different microbial populations (97). Although TPA dioxygenase (*tphA1A2A3*) and dehydrogenase (*tphB*) were not detected in the metagenomic data, degradation could still be occurring using phthalate degradation genes. Metabolic investigation of *Rhodococcus* sp. strain DK17 by Choi and colleagues (98) revealed that the transcription of both the phthalate and terephthalate operons occurs during growth on terephthalate. Additionally, recent biochemical investigations of a purified phthalate dioxygenase from Comamonas testosteroni KF1 showed that phthalate dioxygenase can bind and catalyze the conversions of terephthalate to a dihydrodiol (99). These prior investigations indicate that the phthalate degradation pathway could actively be in use to degrade TPA and terephthalate within the cultures studied here.

Microbial degradation of BPA has been observed previously, but the exact pathway and genes involved in the degradation of BPA are still not entirely understood (51, 52, 74, 75, 78, 100). Previous work has implicated cytochrome P450 (52) and *p*-hydroxybenzoate hydroxylase (*pobA*) (51) genes in the initial transformation of BPA. Benzoate degradation enzymes, the muconate cycloisomerase (*catB*) (Fig. S2D) enzyme, and the benzaldehyde dehydrogenase (*xylC*) (Fig. S2A and B) enzyme have also been implicated in downstream processing of BPA to compounds such as pyruvate or oxaloacetate (51, 52, 75, 78). While cytochrome P450 genes were not detected within the metagenomic data, *pobA* genes were observed within the bulk metagenomic data (Fig. 4C) and within all three *Rhodococcus* MAGs (bins 5, 7, and 12) (Fig. 5). A complete pathway for benzoate degradation was detected in the bulk metagenomic data (Fig. S2C) and within two *Rhodococcus* MAGs (bins 5 and 7) (Fig. 5). A near-complete benzoate degradation pathway (missing cyclohex-1-ene-1-carbonyl-CoA hydratase [*badK*]) was detected within the *Achromobacter* MAG (bin 10) (Fig. 5). Benzaldehyde dehydrogenase (*xylC*) genes were detected within the bulk metagenomic data (Fig. S2A and B) and within two *Rhodococcus* MAGs (bins 5 and 7) (Fig. 5). The *catB* genes were detected within the bulk metagenomic data (Fig. S2D) and within two *Rhodococcus* MAGs (bins 5 and 7), the *Achromobacter* MAG (bin 10), and the *Brevundimonas* MAG (bin 13) (Fig. 5). Organisms within the *Rhodococcus* and *Sphingopyxis* genera have been implicated previously in BPA degradation (75, 78). Metagenomic evidence suggests that members of the *Rhodococcus* genus may be involved in BPA degradation, and while genes implicated in BPA degradation were not detected within the *Sphingopyxis* MAG (bin 4), an organism from the *Sphingopyxis* genus was enriched within cultures grown on BPA (Fig. 3G).

The metagenomic data revealed abundant genes involved in the degradation of alkenes and alkanes that are produced from the pyrolysis of HDPE. Alkane monooxygenases (*alkB*) that can degrade medium (C5 to C11) to long (>C12) chain *n*-alkanes (54, 68) were abundant within the data set and were accompanied by alcohol/aldehyde dehydrogenase genes to completely degrade the alkanes to ω-hydroxy fatty acids (Fig. 4F). Complete alkane degradation pathways to ω-hydroxy fatty acids were observed within all three *Rhodococcus* bins (bins 5, 7, and 12) (Fig. 5). Genes involved in β-oxidation of fatty acids were also highly abundant within the data set (Fig. 4D and E) and within all high-quality MAGs (Fig. 5). Metagenomic data indicate that members of the *Rhodococcus* genus may play an important role in alkane degradation. Additionally, Pseudomonas oleovorans (Fig. 3M) may play an important role in aliphatic hydrocarbon degradation (64) in the cultures, although a high-quality Pseudomonas MAG was not obtained.

Overall, the 16S rRNA gene amplicon and metagenomic sequencing data sets explored here highlight enriched low-diversity microbial communities with a great capacity for the degradation of products derived from the chemical deconstruction of PET, PC, and HDPE plastics (Fig. 3, 4, and 5; Fig. S2). Consistent culture growth and abundant genes relevant to the degradation of TPA, BPA, and aliphatic hydrocarbons derived from HDPE highlight the potential for paired chemical and biological processing of plastic waste (5, 9, 16, 17, 33–37). This work demonstrates that a single microbial community has the potential to process mixed deconstructed plastics paving the way for future work to explore methods for upcycling of mixed deconstructed plastics using a single microbial consortium. Future work should investigate substrate degradation dynamics of these communities and incorporate metatranscriptomic and metabolomic investigations of the cultures to determine active metabolic pathways and metabolic intermediates produced during growth on mixtures of chemically deconstructed PET, PC, and HDPE. Interestingly, the enriched microbial communities shared many common taxa (Table S1 available online at https://doi.org/10.6084/m9.figshare.19126115), revealing that microorganisms with the capacity to degrade plastic depolymerization products may be ubiquitous in a variety of marine, lacustrine, and terrestrial soil environments around the world. Continued efforts should be made to enrich and isolate organisms from the natural environment capable of using deconstructed plastic substrates as a carbon source. If possible, detailed biochemical characterization of enzymes used in these metabolisms should be performed in relevant organisms. These efforts could lead to the discovery of novel enzymes and metabolic pathways for the degradation of plastics and improved paired chemical and biological processing methods that can be industrialized to process plastic wastes in the future.

## MATERIALS AND METHODS

### Chemical deconstruction of PC plastic.

Chemical deconstruction of PC particles was carried out in a 200-mL custom batch reactor, with an aqueous ammonia environment. Aqueous ammonium hydroxide (NH_4_OH; 28 to 30% wt) was provided by Sigma-Aldrich (St. Louis, MO). Different concentrations of NH_4_OH solutions were prepared by mixing the NH_4_OH solutions (28 to 30% wt) with distilled water. A heating tape (HTS/Amptek) was attached to the external wall of the reactor to heat up the batch reactor, and K-type thermocouples were utilized to monitor the temperature of the liquid mixture in the reactor. Five grams of PC particles and 175 mL 28 to 30% NH_4_OH solution were added in the reactor and reacted for 30 min at 120°C. The final liquid product was filtered with Whatman no. 42 filter paper (diameter, 55 mm; pore size, 2.5 μm). Solid products remaining on the filter paper were dried at 55°C in the oven overnight. The liquid product was neutralized to pH 7 using phosphoric acid. Samples were stirred using a magnetic Teflon stir bar and monitored using a pH meter during neutralization.

### Pyrolysis of HDPE plastic.

Pyrolysis of HDPE plastic was performed as described previously by Kulas and colleagues (9) and Byrne and colleagues (17). A brief description of the pyrolysis (101) process can be found in the Appendix. The chemical composition of the liquid pyrolysis oil was published previously by Byrne and colleagues (17).

### Enrichment culturing.

Samples collected from iron-rich stream sediment (Michigamme, MI [46.532, −88.141]), Lake Superior sediment (Bete Grise, MI [47.3723, −87.9529]), vermicompost (Calumet, MI [47.211, −88.553]), and a hydrocarbon seep in the Caspian Sea (39.7455, 50.4806) were used as starting material for enrichment cultures (17). Sediment and compost samples were collected using sterile containers and frozen for preservation. Detailed sample collection methods for Caspian Sea sediments can be found in Mahmoudi et al. (102) and Miller et al. (103).

Enrichment culture media consisted of 90 mL Bushnell-Haas broth (HiMedia Laboratories Pvt Ltd., Mumbai, India) amended with 0.25 g disodium terephthalate, 0.25 g terephthalamide, 2.5 mL BPA, and 0.25 mL of the alkene mixture. Duplicate biological replicates were set up in 250-mL Erlenmeyer flasks using 1 g of soil from the four different locations listed above. Cultures were placed in a shaking incubator at a speed of 200 rpm and at 30°C. Cultures were propagated every 14 days by transferring 10 mL of culture into 90 mL of fresh media. The culture medium composition and conditions remained consistent during the enrichment process. Biomass from cultures were collected after 10 (T5) and 22 (T11) weeks so the microbial community composition of enrichments could be assessed using 16S rRNA gene amplicon sequencing.

### Growth on individual substrates.

Since cultures were enriched on a mixture of substrates, we performed a series of experiments using each individual substrate to determine the substrate preferences of different microbial populations within the cultures. Following 22 weeks (T11) of growth, the culture biomass from each of the eight original cultures was used to inoculate cultures that contained only one of the original substrates on which the cultures were isolated. In addition to the four mixed substrates on which the cultures were isolated (i.e., terephthalamide, disodium terephthalate, BPA, and alkene mixture), cultures were also grown on pyrolyzed HDPE (9). The alkene mixture we used represents some of the model compounds that are related to the compounds produced during pyrolysis, but pyrolyzed HDPE contains a range of carbon chain lengths as well as for each chain length a mixture of the alkane, alkene, and alkadiene compounds, with alkenes dominating. To explore the complexity of the input and the relevance to coupled chemical and biological degradation of plastics, we include pyrolyzed HDPE as an alternative substrate in these experiments.

Single-substrate culture media consisted of 45 mL Bushnell-Haas broth (HiMedia Laboratories Pvt Ltd., Mumbai, India) amended with one of the following: 0.25 g terephthalamide, 0.25 g disodium terephthalate, 2.5 mL BPA, 0.125 mL alkene mixture, or 0.25 mL pyrolyzed HDPE. In addition to the experimental cultures, control cultures transferred into Bushnell-Haas broth with no added carbon substrate and substrate blanks were also set up for each culture and tested substrate. Cultures were grown in 150-mL Erlenmeyer flasks. Cultures were placed on stir plates at room temperature (25°C) and stirred at 200 rpm using sterile Teflon-coated magnetic stir bars. Culture growth was monitored for 10 days, and culture biomass was collected at the end of this period so the microbial community composition of the cultures could be assessed using 16S rRNA gene amplicon sequencing.

### Optical density measurements.

Optical density (OD) measurements were performed directly after inoculation and prior to the collection of biomass from cultures grown on single substrates (104). A description of OD measurement methods can be found in the Appendix.

### Extraction of DNA.

Forty milliliters of culture from each sample was centrifuged at 10,000 × *g* for 20 min at 4°C to pellet the biomass. Culture supernatant was decanted, and biomass pellets were stored at −20°C prior to the extraction of DNA. DNA extractions were performed using the FastDNA spin kit for soil (MP Biomedicals, Irvine, CA) according to the manufacturer’s instructions.

### 16S rRNA gene amplicon sequencing.

DNA extracts were submitted to the Michigan State University (MSU) Genomics Core for amplicon sequencing of the V4 region of the 16S rRNA gene, as described previously (105, 106). A brief description of sequencing methods can be found in the Appendix. DNA extracts from culture blanks and controls were also submitted for sequencing.

### 16S rRNA sequence processing.

Gene amplicon sequences generated by the MSU Genomics Core were processed using mothur v.1.44.3 (107). Paired-end sequence reads were filtered and merged using vsearch v.2.13.3 (108). Sequences with ambiguous bases and more than eight homopolymers were removed using mothur as described previously (106). Chimeric sequences were removed using the mothur implementation of vsearch (108). Processed sequences were clustered into OTUs at a 3% distance threshold using the mothur implementation of the *de novo* distance-based greedy clustering (DGC) algorithm (109). Clustered OTUs were aligned to the SILVA SSURef alignment (v.138), and taxonomic classifications were assigned using mothur.

Following the processing of the 16S rRNA gene sequence data set (6,096 OTUs and 1,044,970 reads), basic contaminant filtering was performed to remove sequences identified as eukaryotes (15 OTUs), archaea (47 OTUs), mitochondria (7 OTUs), chloroplasts (28 OTUs), and unknown (122 OTUs) by SILVA. The final data set used for the analysis consisted of 5,877 OTUs and retained 99.91% of the original reads (1,044,047 reads). Following data set filtering, species taxonomy was assigned using MegaBLAST (110) to identify the closest known relative at the species level for each sequence within the data set.

### Metagenomic sample preparation, sequencing, and data analysis.

Metagenome sequencing on the DNA extracted from the Com-R2-T5 sample was performed by the University of Utah High-Throughput Genomics Core Facility. DNA fragmentation, library preparation, and library normalization were performed using the Nextera Flex DNA library preparation kit (Illumina, San Diego, CA) according to the manufacturer’s instructions. Libraries were evaluated for quality using a bioanalyzer DNA 1000 chip (Agilent Technologies, Santa Clara, CA). Paired-end sequencing (2 × 150 bp) was performed on the Illumina NovaSeq 6000 platform with a NovaSeq S4 reagent kit v1.5 (Illumina). The library was multiplexed and pooled into one lane of Illumina sequencing. Demultiplexing allowing for a single-base mismatch and validation that the sequence lane exceeded the Illumina minimum quality score specifications (greater than 85% of bases higher than Q30 on a 150- × 150-bp sequence run) was performed by the University of Utah High-Throughput Genomics Core Facility.

Paired-end sequences were interleaved using BBMap (111) and assembled using MEGAHIT (112). Reads were mapped using Bowtie2 (113) and reformatted using SAMtools (114). The taxonomy of assembled and mapped contigs was assessed using mmseqs2 (115) and the GTDB reference database (116). Contigs were annotated using Prokka (117). HTSeq (command htseq-count) was used to quantify the number of mapped reads for each gene in the metagenome (118). Protein sequences were further analyzed using GhostKOALA (49) to annotate genes and identify metabolic pathways using KEGG reference databases (119).

Contigs were then binned into categories representing individual taxa using MetaBAT (120), and completeness and contamination were evaluated using CheckM (121). Bins were grouped into “high,” “medium,” “low,” and “Contaminated” categories based on the criteria described previously by Bowers and colleagues (122). Bins were assigned taxonomy using BAT (123), and genes were annotated using Prokka (117). Thirteen metagenome bins were recovered from the Com-R2-T5 metagenome. Six of these bins were of high quality and were selected for further characterization. Protein sequences from each of the high-quality metagenomic bins were further analyzed using GhostKOALA (49) to annotate genes and identify metabolic pathways using KEGG reference databases (119).

### Statistical analyses.

Data exploration and statistical analyses were performed in the R computing environment (124) using the R packages vegan and phyloseq (125, 126). A rarefaction curve was created using the function rarecurve() in the R package vegan (126). Sample richness and Pielou’s evenness were calculated as described previously (63). Bray-Curtis similarities were calculated using the distance function in phyloseq (125), and the PERMANOVA analysis was performed using the function adonis() in the R package vegan (126). Since microbial community data do not follow a normal distribution, Kruskal-Wallis tests followed by *post hoc* Dunn tests were performed using the function dunn.test() from the R package dunn.test (127) to identify microbial populations that were enriched in particular substrates. Box plots displaying OTU abundance and significant Dunn test results and bubble plots displaying the abundance of genes detected in metagenomic data were made using the R package ggplot2 (128).

All R scripts, fasta, excel, and csv files used in analyses are available on FigShare online at https://figshare.com/projects/Microbial_Deconstructed_Plastic_Substrate_Preferences/131882.

### Data availability.

The 16S rRNA gene sequence data and metagenomic sequence data used in this work are publicly available in the NCBI Sequence Read Archive (SRA) under the BioProject identifier (ID) PRJNA849162.
