# Supplementary material for: Deconstructed Plastic Substrate Preferences of Microbial Populations from the Natural Environment
Source: Microbiol Spectr. 2023 Jun 1;11(4):e00362-23. doi: 10.1128/spectrum.00362-23 (PMC10433879; doi:10.1128/spectrum.00362-23)
Supplement: Supplemental file 1 — Supplemental material. Download spectrum.00362-23-s0001.pdf, PDF file, 2.6 MB [file spectrum.00362-23-s0001.pdf]

Supplementary Information for

Deconstructed plastic substrate preferences of microbial populations from the natural environment.

Lindsay I. Putman, Laura G. Schaerer, Ruochen Wu, Daniel G. Kulas, Ali Zolghadr, Rebecca G. Ong, David R. Shonnard, Stephen M. Techtmann

Lindsay I. Putman
Email: liputman@mtu.edu

Stephen M. Techtmann
Email: smtechtm@mtu.edu

S.I. Appendix

S.I. MATERIALS & METHODS:

Pyrolysis of HDPE Plastic:

HDPE was first dissolved in a paraffin wax solvent (123) at a 1:1 ratio to create a liquid feed with a controlled feed of 0.1 kg/hr into the tubular reactor. The pyrolysis reaction occurred at 575 °C with an estimated vapor residence time of 4 seconds (17). A two-condenser system split the pyrolysis vapor into 3 products: a heavy waxy product (>C15), a lighter liquid product (C5-C15), and a gaseous product (C1-C4). The liquid product was then fed to the enrichment cultures.

Optical Density Measurements:

OD measurements were collected using a Genesys 10S UV-Vis spectrophotometer (Thermo Fisher Scientific, Waltham, MA, USA). One milliliter of culture material was pipetted into a two-sided disposable plastic cuvette (VWR International, Radnor, PA, USA), placed into the spectrophotometer, and absorbance was measured at 600 nm. OD_{600} measurements were not collected from cultures grown on terephthalamide. Terephthalamide does not dissolve in water at neutral pH and the presence of substrate solids can interfere with biomass measurements using OD (124). Optical density measurements used to generate Fig. S4 are available on FigShare (https://doi.org/10.6084/m9.figshare.19131782).

16S rRNA Gene Amplicon Sequencing:

Briefly, dual indexed Illumina fusion primers (125) were used to amplify the V4 region of the 16S rRNA gene (515F/806R primers) (100). Product was normalized and pooled using an Invitrogen SequalPrep DNA Normalization Plate and then loaded onto an Illumina MiSeq v2 flow cell and sequenced using a standard 500 cycle reagent kit (100). Quality control, quantitation, and demultiplexing of files was performed as previously described (100).

S.I. RESULTS:

Taxonomic Diversity of Samples Removed from Analyses:

Samples that were discarded from the dataset for further analyses display similar trends. Samples with low sequencing coverage are dominated by the classes Gammaproteobacteria (1.42-95.50%, 58.29% average) and Alphaproteobacteria (0-68.89%, 12.30% average) and contain high abundances of the order Burkholderiales (1.42-95.20%, 54.99% average) (Fig. S3A&B). Control cultures (i.e., cultures without any added carbon substrate) and substrate blanks (i.e., flasks with substrate added to sterile media) yielded little to no biomass (Fig. S4) and are primarily represented in the discarded samples (Fig. S3) with the exception of three samples. One of the control cultures originally inoculated with Caspian Sea sediment grew and sequenced well and substrate blanks containing the alkene mixture and BPA grew and sequenced well (Fig. S5). Similar to observations of the other culture samples were also observed within cultures in the main dataset grown on BPA (Fig. 1B). Similar to observations of the other culture samples discussed thus far (Fig. 1; Fig. S3) Gammaproteobacteria (0.08-82.36%, 53.68% average) and Alphaproteobacteria (6.41-99.22%, 37.71% average) are dominant within these cultures (Fig. S5A). The Caspian Sea control culture appears to be almost completely composed of the order Rhizobiales (99.22%), while the BPA and alkene mixture blank samples are dominated by members of the order Burkholderiales (69.85-73.46%, 71.66% average) (Fig. S5B). Notably, 12.02% of the BPA blank sample consists of organisms from the order Xanthomonadales (Fig. S5B), which with the relative abundance of the top fifty organisms for samples that sequenced poorly (available at https://doi.org/10.6084/m9.figshare.19775377) and control and blank samples that sequenced well (available at https://doi.org/10.6084/m9.figshare.19775401) are published on

FigShare (https://figshare.com/projects/Microbial_Deconstructed_Plastic_Substrate_Preferences/131882).

Taxonomy of Metagenomic Contigs:

Taxonomic assessment of metagenomic contigs reveal that organisms from the families Rhizobiaceae and Pseudomonadaceae dominate the sample (Fig. S5). Organisms within the families Mycobacteriaceae, Burkholderiaceae, and Sphingomonadaceae are also prevalent (Fig. S6). These findings are in agreement with taxonomic information obtained from 16S rRNA gene amplicon sequencing that identified abundant populations of Rhizobiales, Burkholderiales, and Actinobacteria (Fig. 1A&B).

Additional Aromatic Hydrocarbon Metabolic Pathways Relevant to Plastic Degradation:

Near complete pathways for the degradation of toluene and xylene, both common components in gasoline, were identified in the data (Fig. S2A&B). Subunits of the toluene monooxygenase system (*tmoA, tmoB, tmoC, tmoD, tmoE,* and *tmoF*) used in the initial step of toluene degradation and the toluene methyl-monooxygenase (*xylM*) and toluene methyl-monooxygenase electron transfer component (*xylA*) used in the initial step of xylene degradation were missing from both these pathways (Fig. S2A&B). Although the genes for enzymes used in the initial steps of toluene and xylene degradation to (methyl)benzoate were not detected, complete pathways for the downstream degradation of benzoate to catechol (Fig. S2C), and the degradation of catechol to oxoadipate (ortho-cleavage of catechol) and acetyl-CoA or propanoyl-CoA (meta-cleavage of catechol) were identified (Fig. S2D&E). Additionally, two complete dealkylation and monooxygenase pathways for the degradation of aromatic hydrocarbons were identified (Fig. S2F&G), as well as a near complete pathway for β-oxidation ring cleavage (missing cyclohex-1-ene-1-carboyxl-CoA hydratase (*badK*)) (Fig. S2H). Interestingly, the complete trans-cinnamate degradation pathway and a near complete phenylacetate degradation pathway (missing 3-oxo-5,6-didehydrosuberyl-CoA/3-oxoadipyl-CoA thiolase (*paaJ*)) were identified in the metagenomic data (Fig. S2I&J). Trans-cinnamic acid is an important intermediate compound in the biosynthesis of styrene (126) and phenylacetate catabolism is important in the biodegradation of styrene (127).

S.I. DISCUSSION:

16S rRNA Gene Amplicon Sequence Samples Removed from Analyses:

As referenced in the main text, a number of samples (39) sequenced poorly and were removed from the dataset. The taxonomy of these samples was still explored (Fig. S3). Out of those samples 11 were blanks (substrate in media with no culture) or controls (culture in media with no substrate) from the single substrate experiments, which we would expect to remain sterile (blanks) or grow very little (controls). Based on this we would expect very little DNA to be obtained from these samples, which would result in poor sequencing results. The remaining removed samples came from 12 of the iron rich stream sediment cultures, 6 Caspian Sea sediment cultures, and 10 Lake Superior sediment cultures. Notably, all the vermicompost cultures (mixed and single substrate cultures) grew well and sequenced well. Cultures inoculated with the iron rich stream sediment and Lake Superior sediment sequenced poorly under nearly all of the tested conditions. These cultures did not grow as well as the cultures inoculated with vermicompost or Caspian Sea sediment. Since the cultures did not grow dense biomass, less DNA was likely obtained from these samples as well, resulting in poor sequencing results. The poor performance of these cultures could indicate that these two soils did not contain microbial community members capable of utilizing the complex carbon substrates we provided to them.

Role of Ecological Drift in Increasing Culture Microbial Community Dissimilarity:

Ecological drift is defined as random changes in the relative abundance of microbial community members (43) over time. It is one of four primary processes defined as a part of microbial community assembly theory, as originally described by Vellend (43). Changes in the relative abundance of microbial community members due to ecological drift are often related to changes in the birth and death rate of the different microbial community members (43,128). While this is a normal phenomenon in microbial communities, the effects of ecological drift can be enhanced in low diversity communities where the pressure of selection is high and microbial dispersal is low (57,59,60). Our cultures were imposed to strong selection due to the introduction of challenging carbon substrates (57) and were extremely dispersal limited due to complete isolation of cultures within separate flasks. Even biological replicates inoculated with the same starting material could follow different growth and ecological drift trajectories, resulting in highly dissimilar culture communities (mean Bray-Curtis = 0.84). This would be especially pronounced in the low diversity microbial communities that we generated through environmental selection with carbon substrates (57,59,60).

S.I. References

In text-citations in the S.I. text are numbered based on the reference list found in the main manuscript. Please refer to the works cited in the main manuscript.

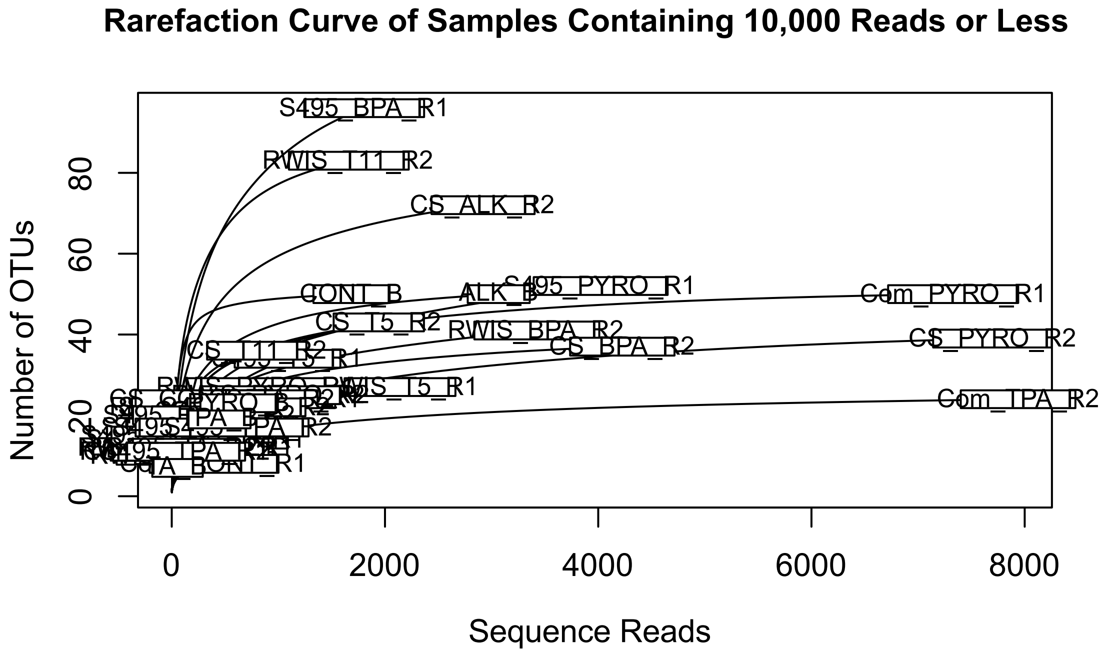

Figure S1. Rarefaction curve plotting samples with 10,000 sequence reads or less. Sequence reads within each sample are plotted against the number of operational taxonomic units (OTUs) detected within each sample.

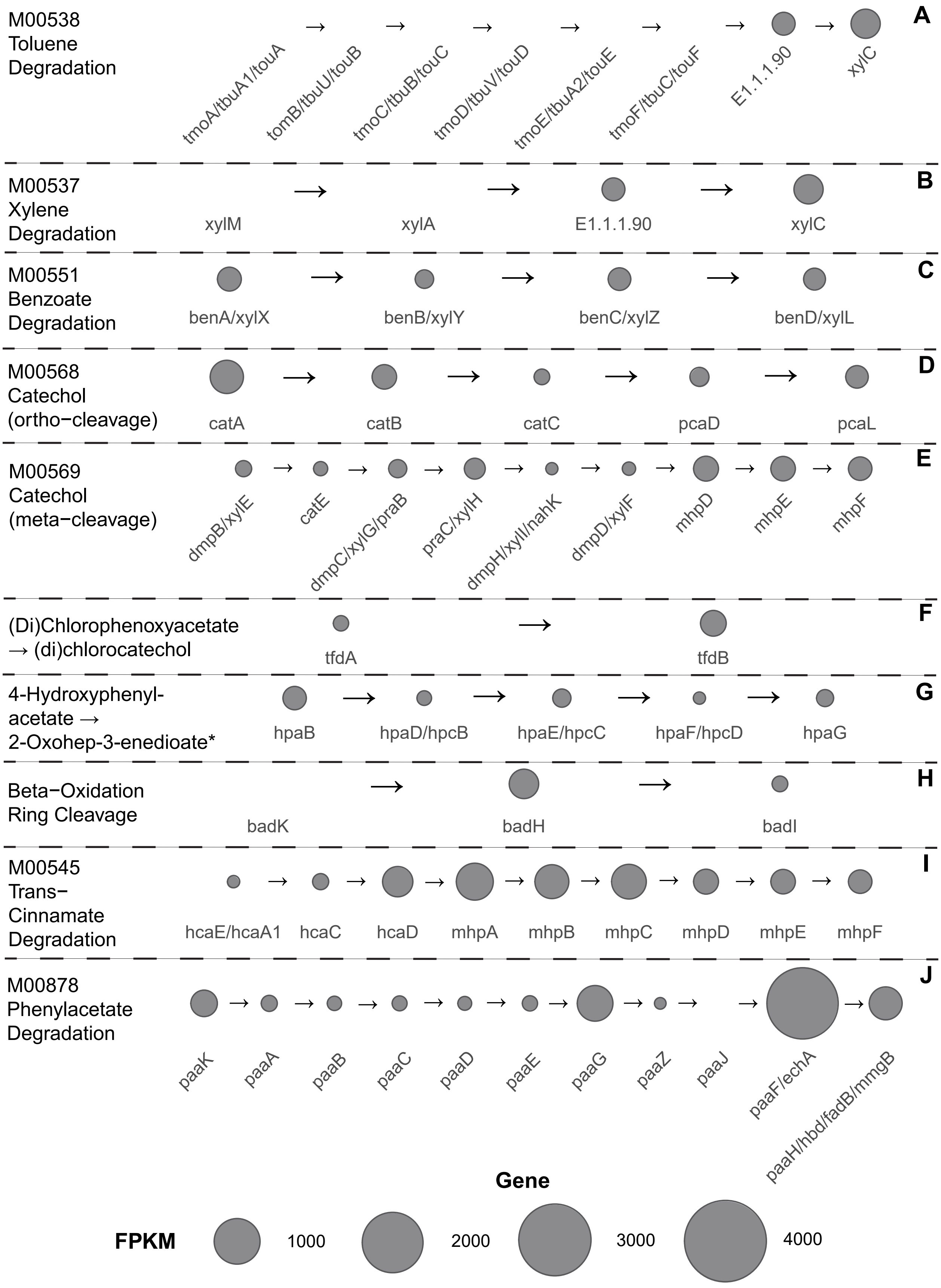

M00538 Toluene Degradation
tmoA/tbuA1/touA
tomB/tbuU/touB
tmoC/tbuB/touC
tmoD/tbuV/touD
tmoE/tbuA2/touE
tmoF/tbuC/touF
E1.1.1.90
xylC
A
M00537 Xylene Degradation
xylM
xylA
E1.1.1.90
xylC
B
M00551 Benzoate Degradation
benA/xylX
benB/xylY
benC/xylZ
benD/xylL
C
M00568 Catechol (ortho−cleavage)
catA
catB
catC
pcaD
pcaL
D
M00569 Catechol (meta−cleavage)
dmpB/xylE
catE
dmpC/xylG/praB
praC/xylH
dmpH/xylI/nahK
dmpD/xylF
mhpD
mhpE
mhpF
E
(Di)Chlorophenoxyacetate → (di)chlorocatechol
tfdA
tfdB
F
4-Hydroxyphenyl-acetate → 2-Oxohep-3-enedioate*
hpaB
hpaD/hpcB
hpaE/hpcC
hpaF/hpcD
hpaG
G
Beta−Oxidation Ring Cleavage
badK
badH
badI
H
M00545 Trans−Cinnamate Degradation
hcaE/hcaA1
hcaC
hcaD
mhpA
mhpB
mhpC
mhpD
mhpE
mhpF
I
M00878 Phenylacetate Degradation
paaK
paaA
paaB
paaC
paaD
paaE
paaG
paaZ
paaJ
paaF/echA
paaH/hbd/fadB/mmgB
J
Gene
FPKM
1000
2000
3000
4000

Figure S2. Bubble plot displaying the summed gene abundance of genes from assembled metagenomic contigs within the KEGG toluene degradation module (A), xylene degradation module (B), benzoate degradation module (C), catechol (ortho-cleavage) degradation module (D), catechol (meta-cleavage) degradation module (E), (di)chlorophenoxyacetate to (di)chlorocatechol pathway (F), 4-hydroxyphenyl-acetate to 2-oxohep-3-enedioate pathway (G), beta-oxidation ring cleavage (H), trans-cinnamate degradation module (I), and phenylacetate degradation module (J). Bubble size corresponds with the detected abundance of each gene, where larger bubbles represent genes detected at high abundances and smaller bubbles represent genes detected at low abundances. Gene abundances are plotted as metagenome fragments per kilobase of predicted protein sequence per million mapped reads (FPKM). *degradation of 4-hydroxyphenyl-acetate (G) occurs via the homoprotocatechuate degradation pathway (KEGG module M0053; hpaD/hpcB, hpaE/hpcC, hpaF/hpcD, and hpaG genes). Genes in each pathway are assembled in order of use in substrate degradation from left to right with arrows in between genes representing the direction of gene use in the pathway.

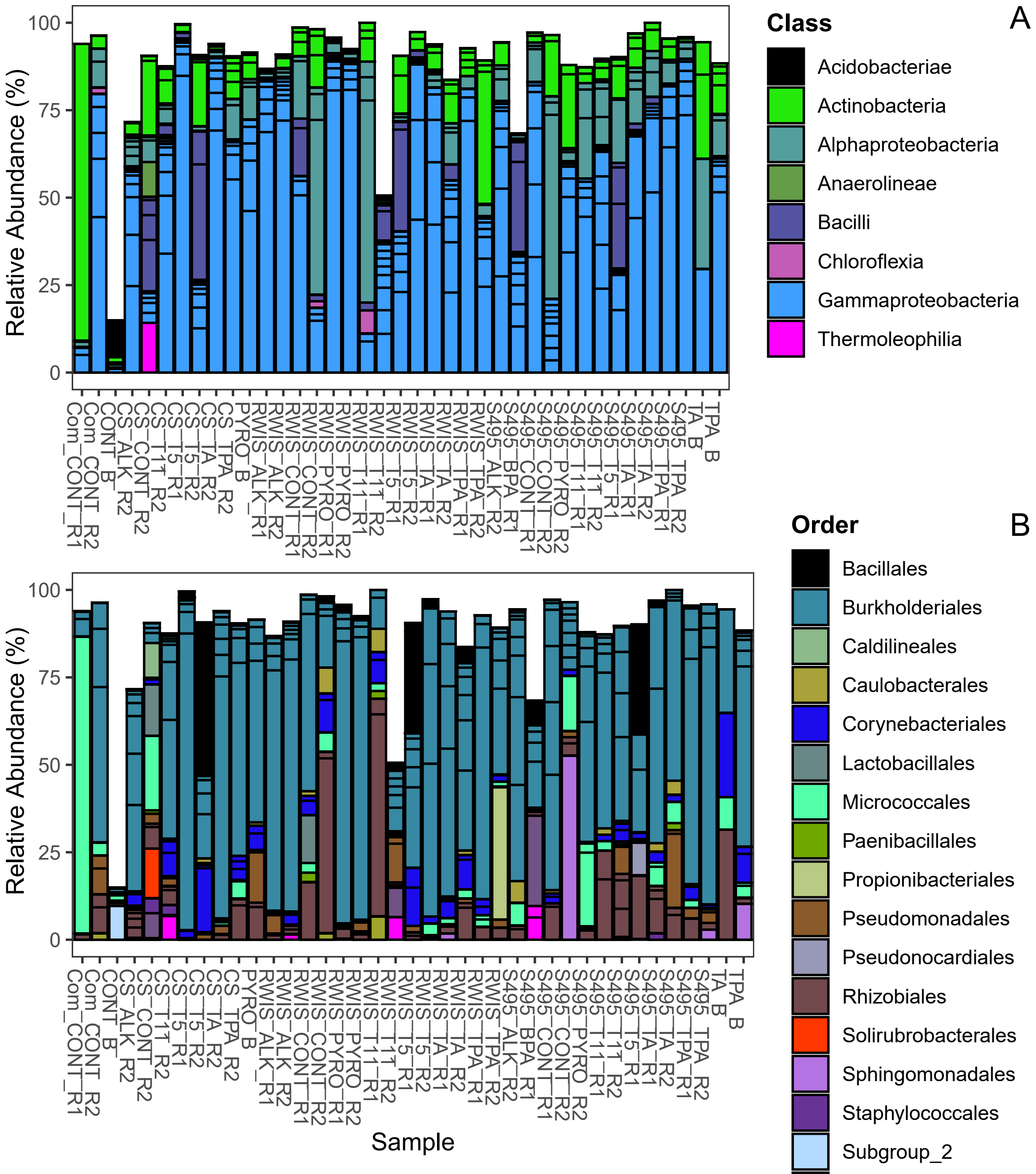
A
Relative Abundance (%)
100
75
50
25
0
Class
Acidobacteriae
Actinobacteria
Alphaproteobacteria
Anaerolineae
Bacilli
Chloroflexia
Gammaproteobacteria
Thermoleophilia
B
Relative Abundance (%)
Sample
Order
Bacillales
Burkholderiales
Caldilineales
Caulobacterales
Corynebacteriales
Lactobacillales
Micrococcales
Paenibacillales
Propionibacteriales
Pseudomonadales
Pseudonocardiales
Rhizobiales
Solirubrobacterales
Sphingomonadales
Staphylococcales
Subgroup_2
Thermicanales
Thermomicrobiales
Xanthomonadales

Figure S3. Stacked bar plots showing the relative abundance of the 50 most abundant OTUs within discarded samples (samples with fewer than 3,000 reads) by taxonomic class (A) and order (B). The 50 most abundant OTUs in discarded samples were determined by assessment of the normalized abundance of OTUs in the 16S rRNA gene amplicon dataset. For cultures grown on mixed substrates sample names used the following convention: "Incolum Source_Transfer Number_Biological Replicate". For cultures grown on individual substrates sample names used the following convention "Inoculum Source_Substrate_Biological Replicate". COM, vermicompost; CS, Caspian Sea sediment; RWIS, iron rich stream sediment; S495, Lake Superior sediment; T5, transfer 5 (10 weeks' time); T11, transfer 11 (22 weeks' time); R1, biological replicate 1; R2, biological replicate 2; ALK, alkene mixture; BPA, deconstructed PC; PYRO, pyrolysis; TPA, terephthalamide; TA, disodium terephthalate; CONT, biological control (no carbon substrate provided); B, blank (biology free media and substrate blanks).

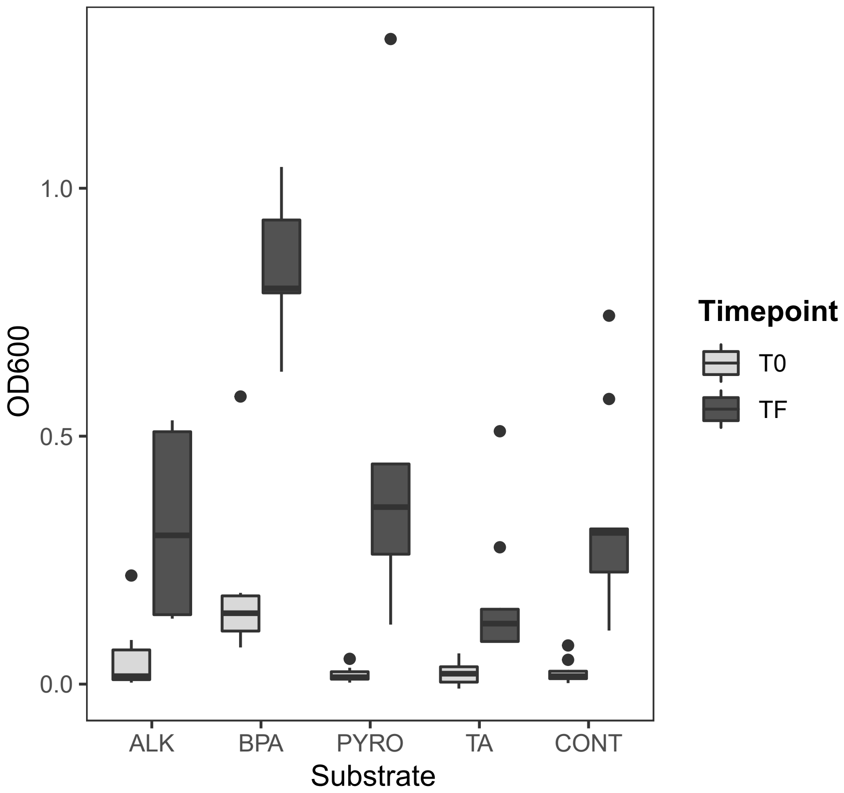

Figure S4. Boxplots showing optical density measurements at 600 nm for cultures grown on different plastic derivative substrates after inoculation (T0) and prior to harvesting biomass (TF). Boxplots display the distribution of data within each category, where the main-colored box displays the interquartile range (25th to 75th percentile) of the data. Solid lines within the boxes represent the median of the data. Whiskers on the plot display the maximum and minimum expected values of the data distribution. ALK, alkene mixture; BPA, bisphenol A; PYRO, pyrolyzed high density polyethylene; TA, disodium terephthalate; CONT, control.

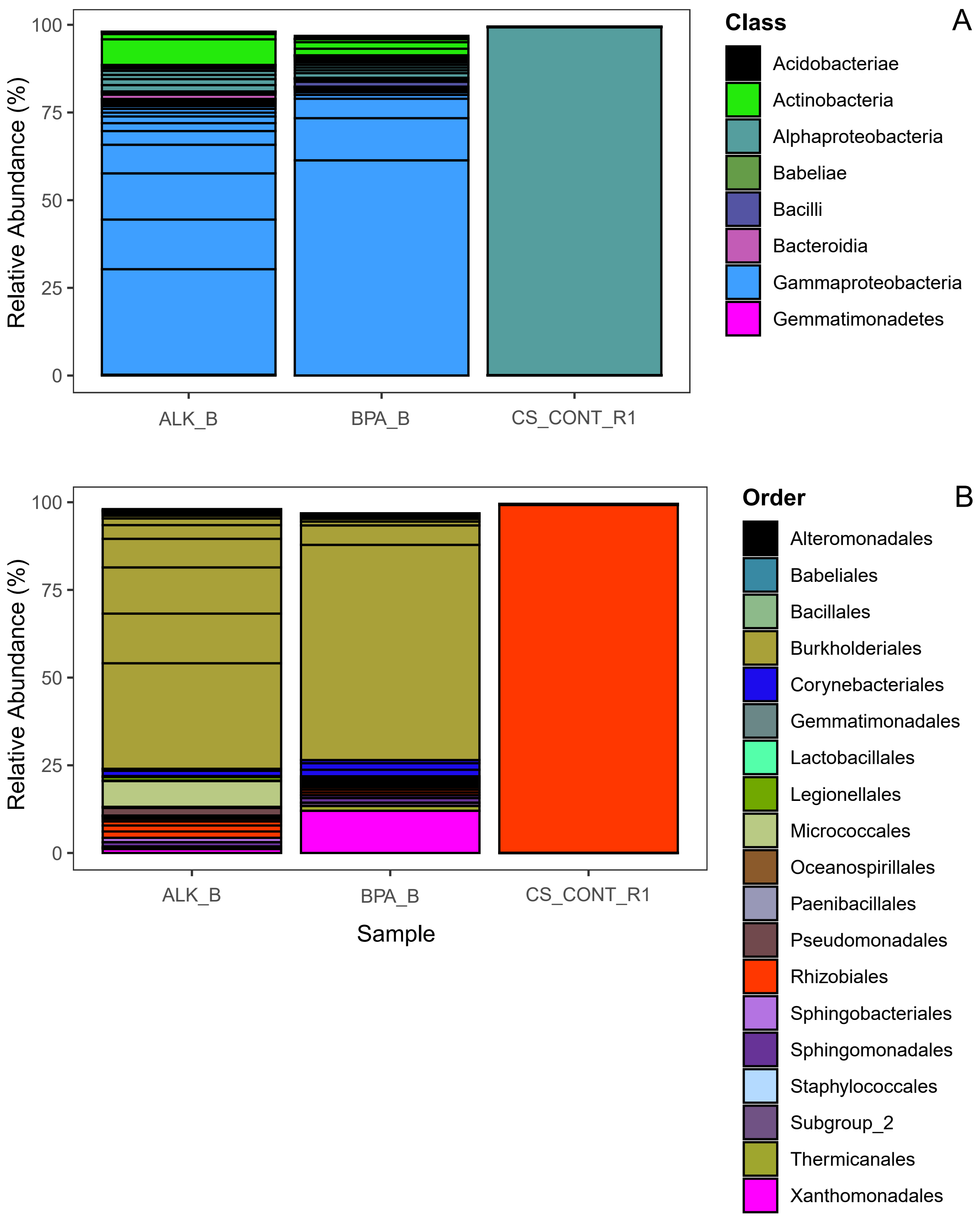
A
Relative Abundance (%)
100
75
50
25
0
ALK_B
BPA_B
CS_CONT_R1
Class
Acidobacteriae
Actinobacteria
Alphaproteobacteria
Babeliae
Bacilli
Bacteroidia
Gammaproteobacteria
Gemmatimonadetes
B
Sample
Order
Alteromonadales
Babeliales
Bacillales
Burkholderiales
Corynebacteriales
Gemmatimonadales
Lactobacillales
Legionellales
Micrococcales
Oceanospirillales
Paenibacillales
Pseudomonadales
Rhizobiales
Sphingobacteriales
Sphingomonadales
Staphylococcales
Subgroup_2
Thermicanales
Xanthomonadales

Figure S5. Stacked bar plots showing the relative abundance of the 50 most abundant OTUs within two blank samples and one control sample that were not discarded for sequencing poorly (samples with fewer than 3,000 reads) by taxonomic class (A) and order (B). The 50 most abundant OTUs in these samples were determined by assessment of the normalized abundance of OTUs in the 16S rRNA gene amplicon dataset. For cultures grown on individual substrates sample names used the following convention "Inoculum Source_Substrate_Biological Replicate". CS, Caspian Sea sediment; R1, biological replicate 1; R2, biological replicate 2; ALK, alkene mixture; BPA, deconstructed PC; CONT, biological control (no carbon substrate provided); B, blank (biology free media and substrate blanks).

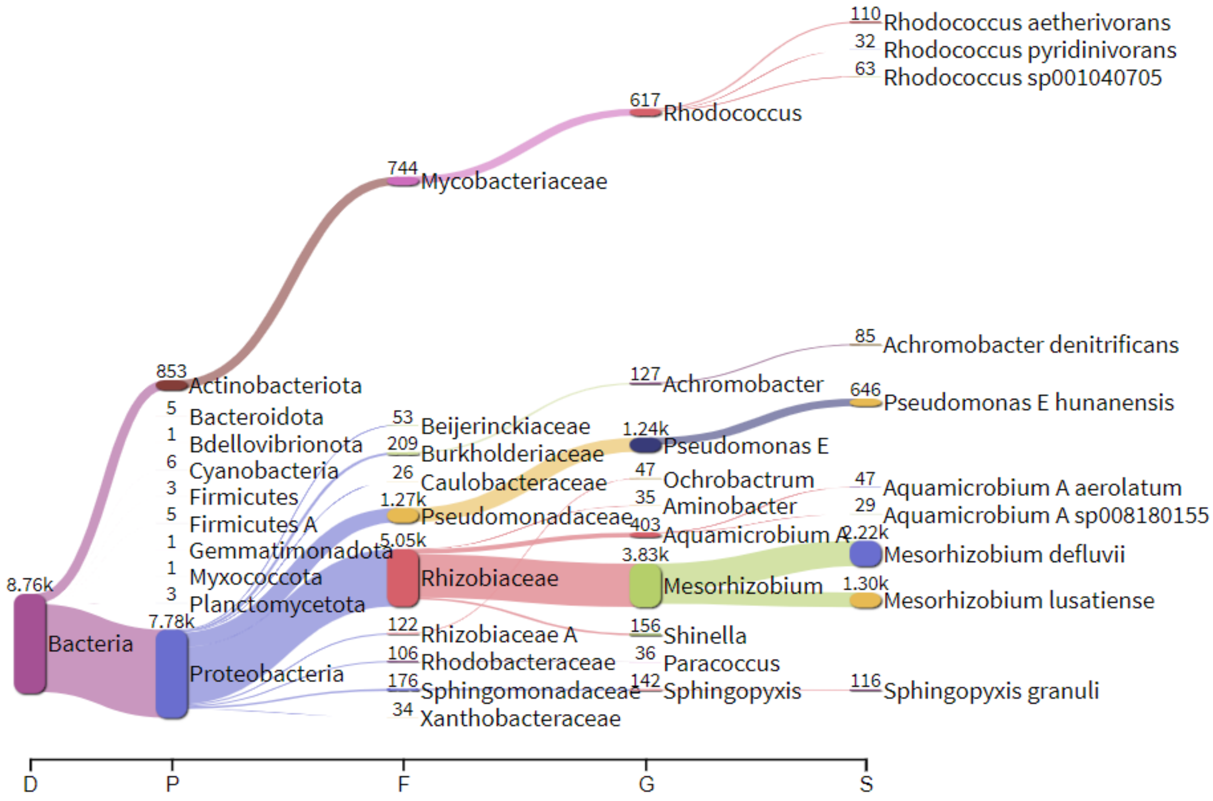

Figure S6. Taxonomic classification of metagenomic contigs visualized using the R package Pavian. Taxonomic classification results in the form of a sankey chart from mmseqs2 were used as input into Pavian. Diagram shows the domain (D), phylum (P), family (F), genus (G), and species (S) of metagenomic contigs and the number of sequence reads associated with each classification.

Table S1 available on FigShare

https://doi.org/10.6084/m9.figshare.19126115

Table S2 - PERMANOVA Results						
**Variable**	**Degrees of Freedom**	**Sum of Squares**	**Mean Squares**	**F Score**	**R2**	**p-value**
Plastic	3.000	1.719	0.573	2.488	0.165	**0.003**
Days	1.000	0.476	0.475	2.064	0.046	**0.046**
Inocula	3.000	3.589	1.196	5.194	0.345	**0.001**
Residuals	20.000	4.606	0.230		0.443	
**Total**	27.000	10.390			1.000	

Table S3 - Kruskal-Wallis Test Results Comparing the Relative Abundance of Dominant OTUs when Grown with Different Single Substrates

Environmental Variable	OTU ID	Species Taxonomy	χ2	Degrees of Freedom	p-value
Plastic Type	OTU_31	Pseudogracilibacillus endophyticus (99.2%)	3.392	2.000	0.180
	OTU_96	Nocardia coeliaca (100%)	7.677	2.000	**0.020**
	OTU_303	Stenotrophomonas maltophilia (100%)	13.582	2.000	**0.000**
	OTU_716	Sphaerobacter thermophilus (85.2%)	1.858	2.000	0.390
	OTU_1032	Paenibacillus thailandensis (98%)	1.417	2.000	0.490
	OTU_1045	Cellulosimicrobium marinum (100%)	1.989	2.000	0.370
	OTU_1054	Oxalicibacterium solurbis (98.8%)	4.228	2.000	0.120
	OTU_1071	Pseudomonas oleovorans (100%)	7.145	2.000	**0.030**
	OTU_1089	Pigmentiphaga litoralis (100%)	3.313	2.000	0.190
	OTU_1098	Ralstonia pickettii (100%)	5.687	2.000	0.060
	OTU_1375	Brevundimonas olei (99.2%)	0.208	2.000	0.900
	OTU_1468	Dietzia lutea (100%)	2.537	2.000	0.280
	OTU_1472	Pelomonas aquatica (100%)	5.859	2.000	**0.050**
	OTU_1574	Gordonia didemni (100%)	18.511	2.000	**0.000**
	OTU_1577	Litorivivens aequoris (81.9%)	10.278	2.000	**0.010**
	OTU_1599	Sphingopyxis granuli (100%)	13.875	2.000	**0.000**
	OTU_1702	Paraburkholderia strydomiana (100%)	12.212	2.000	**0.000**
	OTU_1705	Bordetella tumulicola (100%)	7.616	2.000	**0.020**
	OTU_1709	Brevundimonas diminuta (100%)	1.446	2.000	0.490
	OTU_1724	Sphingobacterium cibi (97.6%)	1.444	2.000	0.490
	OTU_1742	Rhodococcus biphenylivorans (100%)	0.336	2.000	0.850
	OTU_1808	Gemmatimonas phototrophica (95.7%)	0.043	2.000	0.980
	OTU_1836	Rhodococcus soli (100%)	7.643	2.000	**0.020**
	OTU_1951	Sphingomonas montis (100%)	5.957	2.000	**0.050**
	OTU_1967	Mesorhizobium camelthorni (99.2%)	7.682	2.000	**0.020**
	OTU_1972	Ensifer shofinae (99.6%)	2.796	2.000	0.250
	OTU_2227	Altererythrobacter aquaemixtae (99.2%)	8.712	2.000	**0.010**
	OTU_2237	Mesorhizobium norvegicum (98%)	11.786	2.000	**0.000**
	OTU_2838	Rhodopseudomonas harwoodiae (100%)	9.063	2.000	**0.010**
	OTU_2948	Swionibacillus sediminis (97.2%)	1.444	2.000	0.490
	OTU_3075	Castellaniella daejeonensis (99.2%)	2.429	2.000	0.300
	OTU_3146	Hydrogenophaga temperata (99.6%)	6.398	2.000	**0.040**
	OTU_3529	Bosea robiniae (99.6%)	3.168	2.000	0.210
	OTU_4066	Salinibacterium xinjiangense (99.6%)	1.123	2.000	0.570
	OTU_4172	Devosia yakushimensis (98.4%)	2.841	2.000	0.240
	OTU_4237	Arthrobacter crystallopoietes (100%)	2.280	2.000	0.320
	OTU_4676	Aquamicrobium lusatiense (100%)	2.689	2.000	0.260
	OTU_4688	Variovorax defluvii (99.6%)	4.640	2.000	0.100
	OTU_4807	Paenibacillus mucilaginosus (96%)	1.049	2.000	0.590
	OTU_4878	Bradyrhizobium oligotrophicum (97.2%)	3.222	2.000	0.200
	OTU_4886	Kurthia zopfii (100%)	3.635	2.000	0.160
	OTU_4959	Mesorhizobium helmanticense (98.4%)	11.563	2.000	**0.000**
	OTU_5101	Paenibacillus horti (98.4%)	0.084	2.000	0.960

Table S3 - Kruskal-Wallis Test Results Comparing the Relative Abundance of Dominant OTUs when Grown with Different Single Substrates

Environmental Variable	OTU ID	Species Taxonomy	$\chi 2$	Degrees of Freedom	p-value
Plastic Type	OTU_5489	Huakuichenia soli (100%)	2.362	2.000	0.310
	OTU_5655	Luteimonas padinae (100%)	0.576	2.000	0.750
	OTU_5707	Streptococcus thermophilus (100%)	0.247	2.000	0.880
	OTU_5731	Cellulomonas bogoriensis (97.2%)	2.768	2.000	0.250
	OTU_5932	Ochrobactrum intermedium (100%)	0.690	2.000	0.710
	OTU_6016	Methylobacterium phyllostachyos (100%)	14.109	2.000	**0.000**
	OTU_6037	Pseudoxanthomonas mexicana (100%)	0.710	2.000	0.700

Table S4 - Posthoc Dunn Test Results with Benjamini-Hochberg Correction for Significant Kruskal Wallis Tests

OTU ID	Species Taxonomy	Pairwise Comparisons	Z Statistic	p-value
OTU_96	Nocardia coeliaca (100%)	HDPE/PC	-2.738	**0.009**
		HDPE/PET	-0.759	0.224
		PC/PET	1.761	0.059
OTU_303	Stenotrophomonas maltophilia (100%)	HDPE/PC	-3.115	**0.001**
		HDPE/PET	0.486	0.314
		PC/PET	3.282	**0.002**
OTU_1071	Pseudomonas oleovorans (100%)	HDPE/PC	1.888	0.044
		HDPE/PET	2.508	**0.018**
		PC/PET	0.666	0.253
OTU_1472	Pelomonas aquatica (100%)	HDPE/PC	-1.291	0.098
		HDPE/PET	1.320	0.140
		PC/PET	2.420	**0.023**
OTU_1574	Gordonia didemni (100%)	HDPE/PC	-3.781	**2.00E-04**
		HDPE/PET	0.282	0.389
		PC/PET	3.693	**2.00E-04**
OTU_1577	Litorivivens aequoris (81.9%)	HDPE/PC	-2.108	0.026
		HDPE/PET	1.312	0.095
		PC/PET	3.152	**0.002**
OTU_1599	Sphingopyxis granuli (100%)	HDPE/PC	-3.220	**0.001**
		HDPE/PET	0.353	0.362
		PC/PET	3.252	**0.002**
OTU_1702	Paraburkholderia strydomiana (100%)	HDPE/PC	-2.221	**0.020**
		HDPE/PET	1.522	0.064
		PC/PET	3.453	**0.001**
OTU_1705	Bordetella tumulicola (100%)	HDPE/PC	2.174	**0.022**
		HDPE/PET	2.453	**0.021**
		PC/PET	0.355	0.362
OTU_1836	Rhodococcus soli (100%)	HDPE/PC	-2.318	**0.015**
		HDPE/PET	0.398	0.345
		PC/PET	2.477	**0.020**
OTU_1951	Sphingomonas montis (100%)	HDPE/PC	0.985	0.162
		HDPE/PET	2.440	**0.022**
		PC/PET	1.420	0.117
OTU_1967	Mesorhizobium camelthorni (99.2%)	HDPE/PC	2.124	0.025
		HDPE/PET	2.506	**0.018**
		PC/PET	0.450	0.326
OTU_2227	Altererythrobacter aquaemixtae (99.2%)	HDPE/PC	-2.282	**0.017**
		HDPE/PET	0.746	0.228
		PC/PET	2.774	**0.008**

Table S4 - Posthoc Dunn Test Results with Benjamini-Hochberg Correction for Significant Kruskal Wallis Tests

OTU ID	Species Taxonomy	Pairwise Comparisons	Z Statistic	p-value
OTU_2237	Mesorhizobium norvegicum (98%)	HDPE/PC	-2.487	**0.010**
		HDPE/PET	1.110	0.134
		PC/PET	3.304	**0.001**
OTU_2838	Rhodopseudomonas harwoodiae (100%)	HDPE/PC	-3.009	**0.004**
		HDPE/PET	-1.178	0.119
		PC/PET	1.610	0.081
OTU_3146	Hydrogenophaga temperata (99.6%)	HDPE/PC	1.873	0.046
		HDPE/PET	2.328	0.030
		PC/PET	0.509	0.305
OTU_4959	Mesorhizobium helmanticense (98.4%)	HDPE/PC	3.131	**0.003**
		HDPE/PET	2.514	**0.009**
		PC/PET	-0.455	0.325
OTU_6016	Methylobacterium phyllostachyos (100%)	HDPE/PC	-3.020	**0.002**
		HDPE/PET	0.765	0.222
		PC/PET	3.460	**0.001**
